# Supplementary material for: Targeted vibratory therapy as a treatment for proprioceptive dysfunction: Clinical trial in older patients with chronic low back pain
Source: PLoS One. 2024 Jul 19;19(7):e0306898. doi: 10.1371/journal.pone.0306898 (PMC11259273; doi:10.1371/journal.pone.0306898)
Supplement: S1 File — (PDF) [file pone.0306898.s002.pdf]

Research Proposal

＜Title of Research Project＞

Diagnosis and Treatment Device Based on Proprioceptive Approach for the Elderly Patients with Chronic Low Back pain

＜Implementing medical institution＞
National Center for Geriatrics and Gerontology

＜Principal investigator＞
National Center for Geriatrics and Gerontology
Chief of Orthopaedic Surgery
Yoshihito Sakai

May 10, 2021 2nd edition

Table of Contents

1. Clinical Research Implementation System ····························2
2. Clinical Research Background ·······································4
3. Purpose of Clinical Research···8
4. Contents of Clinical Research ·······································9
5. Criteria for selection and exclusion of subjects and discontinuation of clinical research ···11
6. Treatment of clinical research subjects and evaluation of efficacy and safety ··12
7. Reporting of Diseases to an Accredited Clinical Research Review Committee ··15
8. Report of Diseases to the Minister of Health, Labor and Welfare ··16
9. Statistical Analysis ··17
10. Access to original documents ······································18
11. Quality Control and Quality Assurance ·····························18
12. Ethical Considerations ···18
13. Handling and storage of records (including data) ···················20
14.Payment of money and compensation for conducting clinical research ···20
15. Publication of Information on Clinical Research·····················20
16. Duration of Clinical Research··21
17. Explanation to and Consent of Subjects of Clinical Research··21
18. Other matters necessary for the proper conduct of clinical research ···21

Abbreviations and Definitions

List of abbreviations or terms

Abbreviation or terminology	Japanese or English
COP	Center of pressure
RPW	Relative Proprioceptive Weighting ratio
NRS	Numerical rating scale
RDW	Roland-Morris Disability Questionnaire
EQ5D	Euro QOL-5D
GDS	Geriatric depression scale
CSI	Central Sensitization Inventory

1. Clinical Research Implementation System

（1）Research Forms

Research conducted exclusively at the National Center for Geriatrics and Gerontology

（2）Researchers, etc.

Principal Investigator
National Center for Geriatrics and Gerontology　Chief of Orthopaedic Surgery
Yoshihito Sakai
Contact：

Research Representatives
National Center for Geriatrics and Gerontology　Chief of Orthopaedic Surgery
Yoshihito Sakai
Contact：

Researcher
National Center for Geriatrics and Gerontology　Medical Director of Orthopaedic Surgery　Norimitsu Wakao
National Center for Geriatrics and Gerontology　Medical Director of Spine Surgery　Hiroki Matsui

Research contributor
Nagoya Institute of Technology,　Professor of Department of Electrical and Mechanical Engineering, Graduate School of Engineering
Yoshifumi Morita
Aichi Mizuho College,　Assistant Professor of　Institutional Research Center　Kazunori Yamazaki

Research Collaborators
Aichi Prefectural Mikawa Aoitori Medical and Rehabilitation Center for Developmental Disabilities,　Three - Dimensional Motion Analysis Room　Tadashi Ito

Responsible for data management
Nagoya Institute of Clinical Pharmacology　Michiyo Goto
Contact：

Responsible for statistical analysis
Aichi Prefectural Mikawa Aoitori Medical and Rehabilitation Center for Developmental Disabilities,　Three - Dimensional Motion Analysis Room
Tadashi Ito
Contac：

Responsible for monitoring
Nagoya Institute of Clinical Pharmacology　Yoichiro Ban
Contact：

Responsible for audits
None

Research and Development Planning Support Staff
National Center for Geriatrics and Gerontology　Chief of Orthopaedic Surgery
Yoshihito Sakai
Contact：

Coordination and Management Practitioner
（Nagoya Institute of Clinical Pharmacology　Michiyo Goto
Contact：

Persons overseeing the research other than the Principal Investigator and the Principal Investigator
Nagoya Institute of Technology,　Professor of Department of Electrical and Mechanical Engineering, Graduate School of Engineering Yoshifumi Morita
Contact：

（３）Implementing medical institutions and related organizations
National Center for Geriatrics and Gerontology
Address：　7-430, Morioka-cho, Obu city, Aichi Pref.
Contact：

Other Implementing Medical Institutions
None

Related organizations
Nagoya Institute of Technology
Address：Gokiso-cho, Showa-ku, Nagoya city, Aici Pref.
Person in charge：Professor of Department of Electrical and Mechanical Engineering, Graduate School of Engineering　Yoshifumi Morita
Duties：Provide diagnostic and therapeutic equipment for use in this study

Related organizations

Fujita Medical University
Address：1-98, Rakugakubo, Mizukake-cho, Toyoake city, Aichi Pref.
Person in charge：Kazunori Yamazaki
Duties：Measure and analyze proprioceptive sensory function

Related organizations
Aichi Prefectural Mikawa Aoitori Medical and Rehabilitation Center for Developmental Disabilities
Address：9-3 Koyaba, Kouryuji-cho, Okazaki, Aichi Pre
Person in charge：Tadashi Ito
Duties：Measure and analyze proprioceptive sensory function

Nagoya Institute of Clinical Pharmacology（Development contracting organization）
住所：1-2-15, Fukiage, Chikusa-ku, Nagoya city, Aichi Pref.
Address：Junji Ito
Duties：Establishment of case registration systems and databases and preparation of CSRs
Supervision Method：Receive and review reports on the progress of the work.

Research Secretariat
Nagoya Institute of Clinical Pharmacology
Address： 1-2-15, Fukiage, Chikusa-ku, Nagoya city, Aichi Pref.
Person in charge：Junji Ito
Duties ： Oversees research and is responsible for management, including entering into various contracts and managing funds.

Data Center
Nagoya Institute of Clinical Pharmacology
Address：1-2-15, Fukiage, Chikusa-ku, Nagoya city, Aichi Pref.
Person in charge：Michiyo Goto
Duties：Collect data obtained in the study and perform Quality Control of the entire study.

2. Clinical Research Background

（1）Status of Target Diseases in Japan and Overseas

Japan's population is rapidly aging, and in 2015, the aging rate, which is the percentage of the population aged 65 and over, reached a record high of 26.7%, and it is said that the population will continue to age further in the future. In the 2016 survey, low back pain was the leading complaint among men and the second leading complaint among women, and low back pain itself tends to increase with age. In recent years, there have been many reports on the relationship between proprioception and low back pain. Proprioception is an important sense for postural control, including joint

position sense, kinesthesia, mechanics, and perception of the magnitude and timing of muscle contractions, and postural control is believed to be worse in patients with low back pain. Results of foreign studies have shown that the central nervous system of patients with low back pain does not rely on proprioceptive signals from the hip and trunk, but rather on input signals from the lower extremities (Brumagne S, et al. (Brumagne S, et al. Proprioceptive weighting changes in persons with low back pain and elderly persons during upright standing. Neurosci Lett 2004; 366: 366.) In a controlled study of low back pain and non-low back pain patients, researchers experimentally demonstrated that patients with low back pain had inferior balance control in the triceps and superior control in the pollicis propria muscle (Ito T, Sakai Y, et al. (Ito T, Sakai Y, et al. Proprioceptive weighting ratio for balance control in static standing is reduced in elderly patients with non-specific low back Spine 2018; 43(24):1704-1709. (Ito T, Sakai Y, et al. Postural strategy in elderly, middle-aged, and young people during local vibratory stimulation for proprioceptive inputs. Geriatrics 2018; 3(4): E93.) Given the current high prevalence of low back pain in the elderly and the age-related decline in proprioceptive function, it is clear that reduced proprioceptive function can be a contributing factor to low back pain in the elderly.

（２） History and details of standard treatments that have been implemented to date

In terms of treatment aimed at improving proprioceptive function, proprioceptive training, which consists of standing or seated exercises on an unstable supportive base surface such as a balance board or exercise ball, has been shown to be effective. However, such functional training is not generally popular in populations that include a large proportion of elderly people with lower limb muscle weakness and fall-prone tendencies, as well as osteoporotic patients whose falls can easily result in fractures, because the risks are considered to outweigh the benefits.

（3） Current standard of care and treatment outcomes

According to the Low Back Pain Clinical Guidelines 2019 (Revision 2), the only effective treatments with strong evidence for chronic low back pain are serotonin noradrenaline reuptake inhibitors (SNRIs) as pharmacotherapy, weak opioids, and moderate-evidence treatments are non-steroidal anti-inflammatory drugs (NSAIDs), and exercise therapy as the only treatments with moderate evidence. Results of a nationwide randomized trial in Japan comparing oral NSAIDs and exercise therapy with trunk muscle strengthening and stretching showed no difference in low back pain intensity and significant improvement in low back pain-related quality of life (QOL) with exercise therapy. However, there was no clear recommendation for the type of exercise therapy, and no long-term benefit was observed up to 6 months, but no long-term benefit at 1 year.

Regarding adverse events, both drugs and exercise have been reported to cause adverse events, but there have been no reports of rigorous studies of adverse events with exercise therapy.

（4） Issues and uncertainties in the current standard treatment that lead to the need for this study, and novelty and originality of this study compared to previous studies.

This study is a clinical research for the purpose of developing a medical device to accurately diagnose impaired proprioceptive function and to verify whether improvement of impaired proprioceptive function and consequent improvement of low back pain can be achieved. Both the diagnosis and treatment have novelty and originality compared to previous studies.

・About Diagnosis

The frequency band corresponding to the vibration stimulus that is deteriorating is evaluated by continuously applying vibrations of 30-300 Hz to the trunk and lower legs, and measuring the biological responses to these vibrational stimuli as changes in the electrical signals of the foot pressure center using a center of gravity sway meter with closed eyes and standing posture (Figure 1). The frequency band that responds to the vibration stimulus is evaluated by measuring the foot pressure center sway (Fig. 1). Specifically, the following measurements are made.

Assessment of Proprioceptive Function

The measurement procedure consists of a total of two sets of vibration stimuli alternately applied to the gastrocnemius and lumbar multifidus muscles. The measurement conditions are both closed leg and closed eye. The vibration frequency is automatically swept between 30 and 300 Hz, and two types of vibration stimuli are randomly applied in ascending and descending patterns and averaged. 75 seconds are allotted for each measurement, during which time the vibration stimuli are applied at 30 to 300 Hz. A 60-second seated rest period is provided between each set to eliminate residual vibration effects (Figure 2). The transducer is fixed directly to the skin with an elastic band to prevent changes in the vibration site during the test. The Relative Proprioceptive Weighting ratio (RPW) was calculated from the amount of change in the mean position at the center of pressure (COP) in the anteroposterior direction due to vibration stimulation. To obtain additional information on proprioceptive advantage, the relative proprioceptive weighting ratio RPW is calculated by the following equation.

$$RPW = \frac{(\mathrm{abs\ dy\ GS})}{(\mathrm{abs\ dy\ GS})\ +\ (\mathrm{abs\ dy\ LM})}$$

abs dy GS： y-axis deviation of mean COP during triceps stimulation

abs dy LM : y-axis deviation of mean COP during lumbar multifidus stimulation

This formula allows one to evaluate whether the lumbar multifidus or triceps, i.e., trunk or lower extremity, is dominant in postural control; a RPW close to 100% corresponds to lower extremity dominance of the gastrocnemius muscle, while a RPW close to 0% corresponds to trunk dominance.

The method for evaluating eigen sensation in the past was similarly based on vibration stimulation, but it was limited to frequencies around 60 Hz because the muscle spindle is the main eigen sensory receptor to be evaluated. However, in addition to muscle spindles, there are other receptors that control proprioceptive functions, such as Meissner bodies that respond to low frequencies, Furter patches that respond to high frequencies, and muscle spindles that respond to lower frequencies other than 60Hz. The novelty of the diagnostic part of this device is that it can evaluate not only a single frequency but also a continuous wide range of frequencies, thus enabling more detailed evaluation of proprioceptive functions.

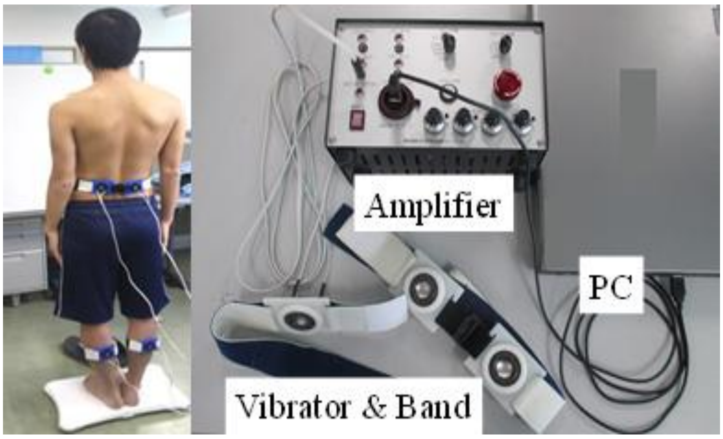

Fig 1. Variable frequency shaker (right) and mounted (left)

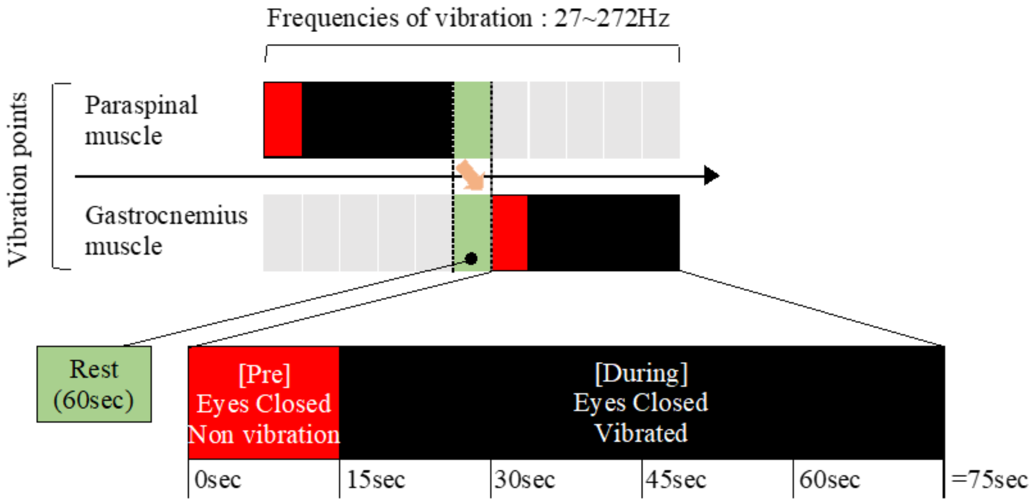

Fig 2. Diagnostic procedure

・Treatment

As mentioned above, there have been previous reports on the use of vibration stimulation to diagnose impaired intrinsic sensory function, but there have been no reports on treatment methods based on this diagnosis. It has been reported in basic research that muscle spindles are activated by vibratory stimulation of muscle tendon parts. If the specific frequency band or intrinsic sensory receptor that is in a state of dysfunction can be identified, it is logical to activate the intrinsic sensory receptor in a state of dysfunction by applying vibratory stimulation to that frequency band. The diagnosis of the frequency band with impaired function itself is novel, and the development of a treatment method that applies specific frequency stimulation to the frequency band with impaired function based on the results of this diagnosis has originality.

(5) Information on pharmaceuticals and other products used in research

1) Name (generic name and trade name) of the drug, etc. concerned

Variable Frequency Vibrators

2) Method and period of use, and basis for setting

The presence or absence of a decrease in the intrinsic sensory function of the trunk or lower leg is confirmed at the medical institution where the research is conducted using the diagnostic method described in (4) above, and vibration stimulation in the frequency band considered to have decreased function is applied to the site of decreased function (trunk or lower leg) in the sitting position for 1 minute three times a day (after breakfast, lunch, and dinner) for 14 days every day at home.

The vibration stimulation will be given to the area with impaired function (trunk or lower leg) in a sitting position for 1 minute three times a day (after breakfast, lunch, and dinner) for 14 days at home.

The equipment will be managed by the medical office by preparing a "Frequency Shaker Loan Management Chart".

Verification of adverse events

After the diagnosis of intrinsic sensory function by the method (4) above, set the frequency for home treatment, give a 2-minute rest after 3 minutes of vibration application of the said frequency stimulus, and again diagnose and evaluate intrinsic sensory function by the method (4) above. The intrinsic function and back pain are evaluated to see if they worsen after a single vibration application. Low back pain will be evaluated using the NRS as a pre-diagnostic and post-diagnostic evaluation.

Verification of treatment efficacy

After the diagnosis of intrinsic sensory function is made at the medical

institution using the method described in (4) above, and the site of dysfunction and frequency band are determined, a portable shaker (Fig. 3) is loaned to the subject, and the subject is asked to apply the shaker at home for one minute per time, three times per day, for two weeks. After 2 weeks, the subject will undergo another diagnostic evaluation of intrinsic function at the medical institution using the method described in (4) above, and the low back pain will be evaluated using NRS and RDQ.

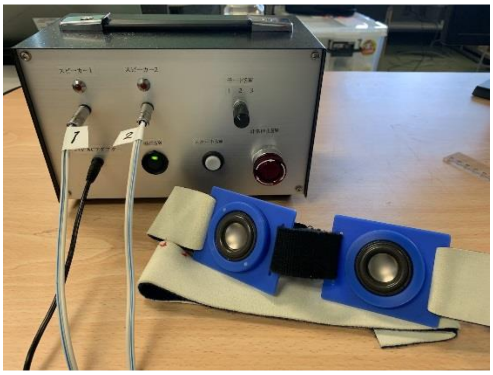
Fig 3. Portable vibrator

Basis for setting

・Duration of vibration stimulation: In a pilot study using 10 volunteers, one of them complained of fatigue in the lower back after 5 minutes of stimulation in all frequency bands. 5 minutes or less was considered desirable because continuous vibration for more than 5 minutes may induce adverse events. Based on a previous study that reported that the recovery of proprioceptive function following vibration stimulation saturates after 1 minute, the duration of therapeutic vibration was set to 1 minute and the frequency to 3 times a day (after breakfast, lunch, and dinner).

（References：Tjernström F et al. Adaptation of postural control to perturbations-a process that initiates long-term motor memory. Gait Posture. 2002 15:75-82, Capicikova N et al, Human posture response to lower leg muscle vibration of different duration. Physiol Res. 2006 55; 129-134.）

・Length of treatment：In previous studies of weak opioid administration for chronic low back pain at the study sites, significant low back pain improvement was achieved after 2 weeks of administration. The duration of treatment was set at 2 weeks in order to expect a therapeutic effect equivalent to that of this pharmacotherapy.

（References：Sakai Y, et al. Pharmacological management of chronic low back pain in older patients: a randomized controlled trial of the effect of pregabalin and opioid administration. Eur Spine J. 2015 24(6) 1309-1317）

3）Target population (age group, gender, disease, etc.)

Patients 65 years of age or older with low back pain that has persisted

for at least 6 months and has not improved after at least 1 month of drug therapy (including NSAIDs, weak opioids, and SNRIs) are eligible. Patients of any gender should have no vertebral fractures (including osteoporotic vertebral fractures, traumatic fractures, or pathological fractures) in the thoracolumbar spine.

4) Clinically significant findings on the efficacy and safety of the drug, etc., obtained from non-clinical studies, other clinical studies, etc.

There is no precedent for the treatment of low back pain using this therapeutic device in both nonclinical and clinical studies. Some previous studies have shown that vibration improves intrinsic sensory function by activating muscle spindles (Burke, 1976; Brumagne, 1989). (1976 Burke, 1989 Brumagne) However, it has been reported that vibration stimulation of a wide range of frequencies in healthy subjects without impaired eigenfunction worsens eigenfunction as a negative effect of vibration stimulation. (2013 Blecher) In this study, the diagnosis of eigensensensory function was made by frequency band using a diagnostic device, allowing for safe treatment by applying vibration only to eigensensensory receptors with impaired function.

5) Known and potential benefits and disadvantages of the use of such medical device

Disadvantage in Diagnosis

When using this device to diagnose proprioceptive function, it is necessary to stand with eyes closed for 60 seconds, which entails the risk of falling during the procedure. Therefore, subjects who cannot hold the standing posture for 60 seconds without assistance will be excluded from the study. In addition, a fall prevention mat will be deployed during the study, and two male assistants (two from the principal investigator or subinvestigator and two from the research associate or collaborator) will always be present to prevent the subject from falling. In more than 200 cases of use of the diagnostic device in the elderly, not a single case of a fall has occurred.

Disadvantages in Treatment

It has already been confirmed that pain and fatigue due to sustained vibration do not occur unless the vibrator is applied for more than 5 minutes. The parts of the transducer that come into contact with the human body are water-resistant, so the possibility of damage is extremely low. Even in the unlikely event of damage, the electrode is covered with an insulating cover to prevent leakage of electricity, thus maintaining safety. The current value that flows to the human body due to electric leakage is less than 5 mA, which is not dangerous to the human body and does not cause any discomfort.

3. Purpose of Clinical Research

In addition to muscle strength and motor function, a decline in proprioceptive sensation is known to be a cause of physical incapacity in

the elderly. The researchers developed a device to accurately diagnose reduced proprioceptive function by utilizing center-of-gravity sway to vibratory stimuli, and reported that reduced proprioceptive function in the elderly is involved in falls and low back pain, and is influenced by the age-related decrease in skeletal muscle mass (sarcopenia). The researchers reported that the decline in intrinsic sensory function in the elderly is related to the occurrence of falls and back pain, and is influenced by the age-related decrease in skeletal muscle mass (sarcopenia). This study aims to improve the intrinsic sensory function of the elderly by applying appropriate vibration stimulation according to the diagnosis of the originally developed diagnostic device, and to examine whether the improvement in intrinsic sensory function leads to improvements in muscle strength, walking function, and low back pain.

4. Contents of Clinical Research

4-1. Primary and secondary endpoints

（1）Primary Endpoint

Variation in NRS scores from pre-treatment (post-diagnosis) to 2 weeks post-treatment with the instrument

（2）Secondary endpoint

Variation in NRS scores before and after instrumented diagnosis and from 2 weeks after instrumented treatment to 2 weeks after the end of treatment

Variation from pre-treatment to 2 weeks post-treatment and 2 weeks post-treatment with instruments for the following items

- RDQscore
- Euro QOL-5D （EQ5D）
- Geriatric Depression Scale（GDS）
- Pain DETECT
- Central Sensitization Inventory（CSI）
- RPWvalue

4-2. Study Design

（1）Intervention

Intervention：Yes

Intervention Description：Vibratory Stimulation in Patients with Low Back Pain

（2）Invasiveness

Invasiveness：Yes

Invasions：Vibratory stimulation of trunk or lower legs

（3）Does the research fall under the category of clinical research on

unapproved or off-label drugs and other medical devices under the Act on the Evaluation of Chemical Substances, Etc.
Yes (use of unapproved medical devices)

（4）Is the research funded by a pharmaceutical company, etc. a clinical research of a drug, etc. of the said pharmaceutical company, etc.?
Not applicable

(5) Method of comparison
Comparison of Before and After Vibration Stimulation in Patients with Low Back Pain

(6) Randomized (randomized) allocation
None

(7) Blindness
Open-label

(8) Clinical Research Procedures
Patients with chronic low back pain that has persisted for more than 6 months and has not responded to drug therapy will be selected through screening and consent for the study will be obtained. After obtaining consent, registration forms will be sent to the data center for case registration. After diagnosis of intrinsic sensory function within one month after consent is obtained, patients will be lent equipment and given home treatment for two weeks. Two weeks after the start of treatment, the patient's back pain will be assessed and treatment will be terminated. Two weeks after that (4 weeks after the start of treatment), the patient's back pain will be assessed again.

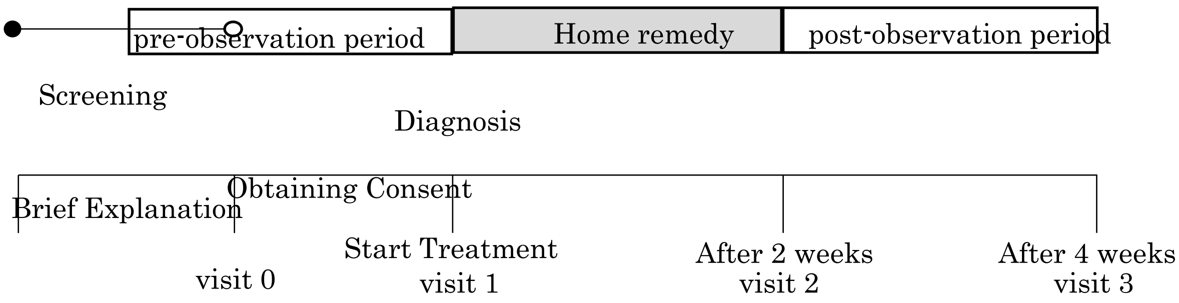

4-3. Drugs and equipment used and management
Portable Vibrator (manufacturing and marketing approval not yet obtained)
The equipment will be managed by Department of Orthopaedic Surgery, National Center for Geriatrics and Gerontologyand loaned

to each subject.
The equipment will be collected after use.

4-4. Expected duration of participation and observation period for clinical research subjects

Within 1 month from obtaining consent to diagnosis (VISIT 1)

2 weeks from start of treatment to end of treatment (data collection at start and end of treatment)

2-week observation period from the end of treatment (data collection 2 weeks after the end of treatment)

4-5. Criteria for discontinuation of clinical research

Clinical research will be terminated in the following cases

1. If it is determined that there is an unanticipated or unacceptable significant risk to the patient
2. When consent is withdrawn by the patient or surrogate
3. If the patient or surrogate requests a change or discontinuation of treatment
4. If the PI or research assistant determines that the continuation of the study is inappropriate due to the occurrence of an adverse event (exacerbation of the underlying disease, worsening of complications, new disease complications, etc.)
5. If you are unable to come to the hospital on the scheduled day due to relocation, etc.
6. Death or serious deterioration in quality of life due to causes not attributable to the study, making it impossible to continue the evaluation
7. In addition to the above, if the PI determines that it is inappropriate to continue the study

When it is decided to discontinue the specified clinical research, the approved Approved Clinical Research Review Committee shall be notified and the Minister of Health, Labour and Welfare shall be notified within 10 days of the discontinuation.

4-6. Identification of the contents that are directly entered in the case report form and should be interpreted as source documents

- Medical records (lab records, medication details, simple radiographs, MRI images, DXA images)
- Numerical Pain Rating Scale （NRS）questionnaire
- Roland-Morris Disability Questionnaire（RDQ）questionnaire
- Euro QOL-5D（EQ5D）questionnaire
- Geriatric Depression Scale（GDS）questionnaire

- Pain DETECT questionnaire
- Central Sensitization Inventory（CSI）questionnaire
- Treatment log

  Results of assessment of proprioceptive function

5. Criteria for selection and exclusion of subjects and discontinuation of clinical research

5-1. Inclusion Criteria

1. Patients who have given their free and voluntary written consent to participate in this study after receiving a full explanation and with full understanding.
2. Patients 65 years of age or older at the time consent is obtained.
3. Patients with chronic low back pain with a NRS value of 3 or higher that has persisted for more than 6 months and has not improved with pharmacological therapy.
4. Patients with no fresh vertebral fracture on lumbar spine X-rays, lumbar spine MRI, etc. performed within 3 months of the start of the study.
5. Patients with a Z score < -1.0 as the criterion for the diagnosis of intrinsic sensory function in Visit 1.

（Basis for setting）

1. To target patients who are able to demonstrate their own free will with sufficient understanding.
2. Because prior studies have shown significant intrinsic sensory function loss in older adults over 65 years of age.
3. Because the treatment targets low back pain of a certain intensity that does not improve with the natural course of the disease. (NRS values of less than 3 are considered to cause less pain.)
4. Because spontaneous relief of back pain may occur during the healing process of vertebral fractures.
5. To study patients with low back pain who have impaired intrinsic sensory function.

5-2. Exclusion Criteria

1. Those who require assistance in standing due to motor dysfunction such as pain or paralysis, or nutritional disorders, and those who cannot hold a standing posture for 60 seconds without assistance.
2. Cerebral infarction, cerebral hemorrhage, brain and spinal cord tumors, central nervous system diseases such as cervical myelopathy and thoracic myelopathy, and muscular diseases such as muscular dystrophy with
3. Any history of vertebral fracture within 1 year of consent income, with or without osteoporosis.
4. Those taking non-steroidal anti-inflammatory drugs (NSAIDs) on a

regular basis.
5. Those with difficulty communicating due to dementia, impaired consciousness, etc.
6. Patients with diseases requiring treatment during the study period.
7. Those with a score of 6 or higher on the Brief Assessment of Risk of Falling in the Elderly (Toba).
8. Patients with psychiatric or psychosomatic disorders who are currently attending a psychiatry department or psychosomatic medicine clinic.
9. Those unable to give consent for this research.
10. Other patients deemed inappropriate by the principal investigator or subinvestigator.

(Basis for setting)
1. Insufficient diagnosis of intrinsic sensory function cannot be performed prior to the start of treatment.
2. Insufficient diagnosis of intrinsic sensory function cannot be performed prior to the start of treatment.
3. Because spontaneous relief of back pain may occur during the healing process of vertebral fractures.
4. If the patient has severe back pain, the pain may worsen during the examination.
5. Because it may not be possible to properly assess low back pain.
6. Due to concerns about the impact of disease treatment interventions during the study period.
7. Because of the risk of falling during the inspection
8. Because psychogenic back pain cannot be ruled out.
9. The setting was established to ensure the safety of the research subjects.
10. The setting was established to ensure the safety of the research subjects.

5-3. Criteria for discontinuation of clinical research for each subject

If the Principal Investigator and the Research Assigning Physician determine that it is impossible to continue treatment with the device for any of the following reasons, treatment with the device for the Research Subject will be discontinued. In such a case, the investigator will explain to the research subject that the treatment will be discontinued and the reason for such discontinuation, and necessary treatment and observation will be carried out.

1. When it is determined that the continuation of treatment with the device is not desirable due to the occurrence of an adverse event.
2. If you are found to be ineligible as a research subject after treatment with the device.
3. If the subject of the clinical research withdraws consent to

participate in the clinical research.

(Basis for setting)
1～2. The settings were established to ensure the safety of the subjects in the clinical study.
For 1, this includes the addition of nonsteroidal anti-inflammatory drugs (NSAIDs) due to worsening back pain.
3. It was established to ensure the ethicality of the subjects of clinical research.

6. Treatment of clinical research subjects and evaluation of efficacy and safety

6-1. Treatment Schedule（Table1）

Duration of treatment by device

Vibration stimulation of the frequency set for the treatment area determined by the diagnosis of intrinsic sensory function, 3 times a day (morning, noon, and after dinner), 1 minute per session.

Observation period

2 weeks after the treatment period with the device

Visiting Schedule

Date of registration and diagnosis of intrinsic sensory function after obtaining consent (day before the start of treatment), 2 weeks after the start of treatment with the device, and at the end of the observation period (4 weeks after the start of treatment)

Dietary restriction

None

Table 1. Visit and Treatment Schedule

	Visit 0	Visit 1		Visit 2		Visit 3
		Day0	Day1 ~13	Day14	Day15 ~27	Day28
		Diagnosis	Treatment	Evaluation	Post-observation	
Visit	●	●		●		●
Informed Consent	●					
Case Registration	●					
Patient Background	●					
Height and Weight		●				
Check concomitant medications	●	●		●		●
Temperature		●		●		●
Blood Pressure		●		●		●
Skin Condition	●	●		●		●
Standing and walking status	●	●		●		●
LBL Evaluation	●	●		●		●
Numerical Rating Scale (NRS)		●*		●		●
RDQ score		●		●		●
EuroQOL-5D (EQ5D)		●		●		●
Geriatric Depression Scale (GDS)		●		●		●
Pain DETECT		●		●		●
Central Sensitization Inventory (CSI)		●		●		●
Proprioceptive diagnosis: RPW value		●		●		●
Frequency vibration stimulation		●				
Vibratory therapy**		●	●	●		
Treatment effect (RPW value)				●		●

* The NRS evaluation in visit 1 is performed twice, before and after instrumental diagnosis.

** Vibratory treatment will be given 3 times a day (morning, noon, and after dinner) for a total of 42 times. visit1 will be set in the evening, and the vibration given after diagnosis in visit1 will be the initial treatment, and the noon treatment on Day14 in the home treatment will be the final treatment.

Numerical Rating Scale (NRS) : Assessment of back pain intensity
Roland-Morris Disability Questionnaire (RDQ) : Assessment of ADL disability due to low back pain
Euro QOL-5D (EQ5D) : Quality of Life Assessment of the Elderly
Geriatric Depression Scale (GDS) : Assessment of depressive tendencies

Pain DETECT：Evaluation of neuropathic pain
Central Sensitization Inventory（CSI）：Evaluation of central sensitization

6-2. Concomitant Restricted (Prohibited) Drugs and Concomitant Restricted (Prohibited) Therapies

Restricted (prohibited) concomitant medications: None, but no new drug therapy for low back pain or other pain should be started from the time consent is obtained until the end of the observation period, nor should any changes in medications used prior to obtaining consent be made.

Restricted (prohibited) concomitant therapy: None, but no new therapy (e.g., exercise, physical therapy) for low back pain or other pain must be initiated, and no change in therapy (e.g., exercise, physical therapy) must be made before consent is obtained, from obtaining consent to the end of the observation period.

6-3. Other Agreements

None

6-4. Evaluating Efficacy

（1） Primary endpoints

Variation in NRS score from before to 2 weeks of treatment with the device

（2） Secondary endpoint

Variation in NRS scores before and after instrumented diagnosis and from 2 weeks after instrumented treatment to 2 weeks after the end of treatment

Variation from pre-treatment to 2 weeks post-treatment and from 2 weeks post-treatment to 2 weeks post-treatment with the following instruments

・RDQ score
・Euro QOL-5D （EQ5D）
・Geriatric Depression Scale（GDS）
・Pain DETECT
・Central Sensitization Inventory（CSI）
・RPW value

The NRS is an assessment measure of the intensity of low back pain. It is performed at the time of intrinsic sensory diagnosis (VISIT 1), 2 weeks after treatment with the device (VISIT 2), and 2 weeks after the end of treatment (VISIT 3).

The RDQ score is an ADL assessment index due to low back pain.

It is administered at the time of intrinsic sensory diagnosis (visit 1), 2 weeks after treatment with the device (visit 2), and 2 weeks after the end of treatment (visit 3).

The Euro QOL-5D (EQ5D) is a quality of life assessment index for the elderly. It is administered at the time of intrinsic sensory diagnosis (visit 1), 2 weeks after treatment with the device (visit 2), and 2 weeks after the end of treatment (visit 3).

The Geriatric Depression Scale (GDS) is a measure of depression. It is administered at the time of the eigensensensory diagnosis (visit 1), 2 weeks after treatment with the instrument (visit 2), and 2 weeks after the end of treatment (visit 3).

Pain DETECT is a measure of neuropathic pain. It is performed at the time of intrinsic sensory diagnosis (visit 1), 2 weeks after treatment with the device (visit 2), and 2 weeks after completion of treatment (visit 3).

The Central Sensitization Inventory (CSI) is an assessment measure of central sensitization. It is administered at the time of the intrinsic sensory diagnosis (visit 1), 2 weeks after treatment with the device (visit 2), and 2 weeks after the end of treatment (visit 3).

The RPW value is an evaluation index of intrinsic sensory function. It is performed at the time of eigensensensory diagnosis (visit 1), 2 weeks after treatment with the device (visit 2), and 2 weeks after the end of treatment (visit 3).

See "9. Statistical Analysis" for methods of analysis.

6-5. Safety Assessment

（1）Physical findings

Check skin conditions (itching, redness, discomfort, etc.)

（2）Vital signs

Blood pressure and temperature measurement

（3）Low back pain evaluation

Worsening of back pain during equipment use

身 Physical findings and vital signs will be evaluated at the time of diagnosis of intrinsic function and prior to examination at 2 weeks after treatment and 2 weeks after completion of treatment (VISIT 1, 2, 3).

The examination should be stopped if the patient's body temperature is 37.5° C or higher or systolic blood pressure is 180 or higher. The examination shall also be discontinued if back pain worsens during

diagnosis and treatment with the device. If the NRS of low back pain worsens before and after a single stimulation, participation in this study will be terminated. In the event of illness or discontinuation of testing after the start of treatment with the device, the principal investigator will examine the patient and guide him/her to the appropriate department for consultation. In the case of other unknown diseases that are suspected to be related to the study, the patients will be followed up until the study is terminated or their symptoms improve.

7. Reporting of Diseases to an Accredited Clinical Research Review Committee

When the Principal Investigator becomes aware of any of the following matters, he/she shall report the matter to the Administrator of the implementing medical institution within the time period specified for each, notify the Principal Investigator, and promptly provide information to the Principal Investigators at each facility to that effect. The principal investigators at each site will promptly report the details of such information to the administrator of the implementing medical institution.

The principal investigator reports to the administrator of the implementing medical institution, and then reports the information to an accredited clinical research review committee.

A Outbreaks of the following diseases, etc., which are suspected to be caused by the implementation of specific clinical research using unapproved or off-label drugs, etc., and which cannot be predicted

Seven days

I Death

II Diseases that may lead to death, etc.

B Outbreaks of the following diseases, etc., which are suspected to be caused by the implementation of specific clinical research using unapproved or off-label drugs, etc. (excluding those listed in A)

15 days

I Death

II Diseases that may lead to death, etc.

C Outbreaks of the following diseases, etc., which are suspected to be caused by the implementation of specific clinical research using unapproved or off-label drugs, etc., and which cannot be predicted (excluding A)

15 days

I Diseases requiring hospitalization or extended hospitalization for treatment

II Disability

III Diseases, etc. that may lead to disability

Ⅳ I to Ⅲ, as well as diseases that are as serious as death and diseases that may lead to death, etc.
Ⅴ Congenital diseases or anomalies in later generations

In addition, illnesses, etc. (known, non-serious events, excluding those listed above) suspected to have resulted from the conduct of specific clinical research should be reported when making periodic reports to an accredited clinical research review committee.

If a principal investigator becomes aware of the occurrence of a defect in a medical device (or regenerative medicine product) used in the specified clinical research that may cause any of the diseases listed below, the principal investigator shall report to the administrator of the medical institution within 30 days of becoming aware of such a defect, and shall then notify the principal investigator and promptly provide information to the principal investigators at each facility. The principal investigator at each site shall promptly provide information to the principal investigator at that site.

The principal investigator at each site will promptly report the details of such information to the administrator of the site.

The principal investigator reports the information to the administrator of the site and then to an accredited clinical research review committee.

A Death
B Diseases that may lead to death, etc.
C Diseases requiring hospitalization or extended hospitalization for treatment
D Disability
E Diseases, etc. that may lead to disability
F C to E, and diseases which are as serious as death and diseases which may lead to death.
G Congenital diseases or anomalies in later generations

8. Report of Diseases to the Minister of Health, Labor and Welfare

When the principal investigator becomes aware of any of the following matters, he/she shall report it to the Minister of Health, Labour and Welfare within the time period specified for each.

A Outbreaks of the following diseases, etc., which are suspected to be caused by the implementation of specific clinical research using unapproved or off-label drugs, etc., and which cannot be predicted.

<u>7 days</u>

Ⅰ Death
Ⅱ Diseases that may lead to death, etc.

B　Outbreaks of the following diseases, etc., which are suspected to be caused by the implementation of specific clinical research using unapproved or off-label drugs, etc., and which cannot be predicted.

15 days

I　Diseases requiring hospitalization or extended hospitalization for treatment

II　Disability

III　Diseases, etc. that may lead to disability

IV　I to III, as well as diseases that are as serious as death and diseases that may lead to death, etc.

V　Congenital diseases or anomalies in later generations

9. Statistical Analysis

9-1. Population to be analyzed

The largest analysis set (Full Analysis Set: FAS) will be all subjects who have received at least one treatment with the device and who have been followed for at least one observation. However, subjects who do not meet the eligibility criteria will be excluded.

The Per Protocol Set (PPS) will be those subjects in the FAS who do not deviate significantly from the protocol and can be evaluated for the primary endpoint.

The target population for safety analysis is all subjects who have received at least one treatment with the device.

9-2. Statistical Analysis Methods in Efficacy

The primary endpoint, the NRS score at 2 weeks, will be analyzed using a test of the difference between the means of the two corresponding groups in comparison to the value before the start of treatment (after diagnosis). The primary analysis will be performed using the FAS; if both the pre-treatment and 2-week post-treatment NRS scores in the FAS are not normal, a Wilcoxon's signed rank sum test will be performed. In addition, to evaluate the effect of maintenance of treatment after completion of treatment, the values at 4 weeks will be analyzed using the test of difference of means of the two groups with a corresponding comparison with the values at 2 weeks; if either the NRS scores at 4 weeks or 2 weeks are not normal, a Wilcoxon's signed rank sum test will be performed.

The secondary endpoints, RDQ, EQ5D, GDS, Pain DETECT, and CSI scores and RPW values, are analyzed using the PPS, with values at 2 weeks analyzed using the test of difference of means for the two groups in comparison with values before the start of treatment, but if either of the scores in the PPS are not normal, the Wilcoxon

signed rank sum test is used. If either one of the scores in the PPS does not have normality, a Wilcoxon signed rank sum test is performed. In addition, to evaluate the effect of treatment maintenance after the end of treatment, the values at 4 weeks are analyzed using the test of the difference of means of the two groups with the corresponding comparison with the values at 2 weeks, and if either of the scores for the secondary endpoints at 4 weeks or 2 weeks are not normal, a Wilcoxon signed rank sum test is performed.

9-3. Statistical Methods in Intermediate Analysis

No intermediate analysis will be performed, and statistical analysis will be performed after the study is completed using the 9-2 methodology.

9-4. Planned number of cases for analysis and rationale

Planned Analysis Cases

Entire study 30 cases

(Basis for Establishment of Scheduled Cases)

Using data on low back pain NRS in a weak opioid trial (N=30) in elderly (65 years and older) chronic low back pain patients, we hypothesized that the improvement would be equivalent to that of clozapine treatment. The difference in mean NRS values before and after treatment (2 weeks) was 1.7, with a common standard deviation of 3.1, an alpha error of 0.05, and a power of 0.08, using "Easy-R" (EZR) (Saitama Medical Center, Jichi Medical University, Saitama, Japan). University, Saitama, Japan) was used to calculate the sample size for comparison of the means of the two groups.

(Reference：Sakai Y, et al. Pharmacological management of chronic low back pain in older patients: a randomized controlled trial of the effect of pregabalin and opioid administration. Eur Spine J. 2015 24(6) 1309-1317)

9-5. Criteria for discontinuation of clinical research

5.5 Criteria for Discontinuation of Clinical Research

9-6. Procedures for handling missing, rejected and abnormal data

Deficient NRS scores at 2 and 4 weeks shall be rejected.

From the treatment diary, those with less than 50% of the number of procedures performed shall be rejected.

9-7. Procedures for changing the original statistical analysis plan

If any changes are made to the original statistical analysis total, the research protocol shall be revised and described in the Supervisory Report for the clinical research.

10. Access to original documents

The principal investigator shall cooperate directly in the monitoring related to the clinical research and in the investigations by the accredited clinical research review committee and domestic and foreign regulatory authorities, with respect to the source documents and other materials (including the details of the agreement with the fund provider). The investigator shall cooperate with direct access to all clinical research-related records of the

11. Quality Control and Quality Assurance

11-1. Monitoring Methods

Monitoring shall be conducted in accordance with the Monitoring Procedures.

12. Ethical Considerations

12-1. Burden on research subjects, anticipated risks

The following risks are anticipated by participating in this study.

・Fall

Because the assessment of proprioceptive function includes testing performed in a standing position with closed eyes, there is a risk of falling during the test.

・Skin disorder

The treatment device is applied to the back of the lumbar region or the posterior aspect of the lower leg for 5 minutes to provide vibration stimulation, which may cause redness and itching as a skin disorder due to contact with the vibrator.

・Damage to equipment and electrical leakage

The parts of the transducer that come into contact with the human body are water-resistant and extremely unlikely to be damaged during use. Even in the unlikely event of damage, the electrodes are covered with an insulating cover to ensure safety without risk of electrical leakage. The current flowing to the human body due to leakage is less than 5 mA, so there is no possibility of danger to the human body, and no unpleasant sensation is expected to occur.

In addition, participation in this study may result in the following burdens

・Mental anguish

This may occur if you feel compelled to cooperate with this study. If this occurs, explain well in advance that you may discontinue participation in the study immediately.。

・Hospital visit for evaluation

A total of three visits are required: at the time of diagnosis of

proprioception (visit 1), two weeks after treatment with the device (visit 2), and two weeks after the end of treatment (visit 3). Considering the time required and the financial burden of using transportation, a fee of 2,000 yen per visit will be paid as a burden reduction fee. (Payment will be made by bank transfer or QUO card.)

12-2. Projected Profits

Participation in this study is expected to improve activities of daily living (ADL) and quality of life (QOL) by improving low back pain. In addition, the improvement in proprioceptive function is expected to improve the ease of walking and decrease the tendency to fall. Even if there is no direct benefit to the research subjects, the research results may contribute to future medical advances.

12-3. Overall assessment of burdens, risks, and benefits

• Fall

Regarding the subject's falls during the examination, those who are considered to have severe fall-prone tendencies with a score of 6 or higher after a simple fall risk assessment of the elderly (Toba) by a physician during the screening process will not be included in the criteria. Even if the subject has a mild susceptibility to falls, the device may improve the intrinsic sensory function of the subject, and therefore, the device can be expected to improve the susceptibility to falls.

• Skin disorder

During screening, ask about skin diseases, allergies, and susceptibility to irritation from topical products, etc. Explain that any possible skin problems are temporary and will improve with interruption of testing and treatment. Explain that any skin problems that may occur are temporary and will improve with interruption of testing and treatment, and that the patient may discontinue the study immediately if he or she feels distress.

• Damage to equipment and electrical leakage

Explain that damage to the equipment will not occur unless it is subjected to a severe external shock, and that even in the unlikely event of an electrical leakage, no current will be generated that could harm the human body.

We will explain that even taking into account the possibility of the above-mentioned risks, the treatment with this device may bring about the benefit of improvement of back pain.

Regarding the mental burden and the burden of hospital visits, since the subjects are selected from patients who are currently undergoing orthopedic surgery for treatment of back pain, it is assumed that a relationship of trust has been established with the

principal investigator or subinvestigator as the attending physician. Since the subjects are motivated to improve their back pain, we assume that the psychological burden will not be too great, taking into account the details of the study. In addition, we believe that the subjects' location will be taken into consideration, and that eliminating the financial burden of hospital visits will substantially reduce the burden on the subjects.

12-4. Measures to minimize burden and risk

As a precaution against falls, always have at least two researchers or assistants involved in the measurement, excluding the instrument operator, present during the examination when the patient is standing, and support the patient in case of a fall, especially when the patient's eyes are closed. Place a fall prevention mat on the floor around the measurement device to prevent health hazards in the event of a fall. Since there is a possibility that the patient may feel fatigue during or after the measurement, we will take measures such as ensuring that the patient takes sufficient rest and drinks enough water, etc. If the subject indicates that he/she is unable to continue the study, we will immediately terminate the study at that time.

13. Handling and storage of records (including data)

The following materials obtained in this study will be stored under the responsibility of the principal investigator, with data password-protected and paper materials stored securely in a locked cabinet. The storage period is 5 years from the date of completion of the specific clinical research. When destroyed, the materials should be shredded or incinerated in a manner that makes them unrecoverable.

1) The research protocol, implementation plan, documents pertaining to the explanation to the subjects of the specified clinical research and their consent, summary report, and other documents prepared by the principal investigator pursuant to the provisions of this Ministerial Ordinance, or copies.
2) Documents received from accredited clinical research review committees pertaining to review opinion services.
3) Monitoring Documents
4) Original documents, etc.
5) Agreement for the Conduct of Specific Clinical Research
6) Documents describing the outline of drugs, etc. to be used in the specified clinical research

Samples and information obtained in this study will be managed in a form in which individuals cannot be identified (subject numbers), and will be provided to the Mikawa Aoitori Medical and Rehabilitation Center

in Aichi Prefecture, the facility to which the person responsible for statistical analysis belongs, for further analysis. The data provided will be password-protected and paper materials will be stored securely in a locked cabinet at the facility to which the data are provided. The storage period is five years from the date of completion of the specific clinical research. When data is destroyed, it should be processed in a form that cannot be restored (e.g., shredded or incinerated).

14. Payment of money and compensation for conducting clinical research

If, in the course of conducting this research, a health hazard occurs to a research subject during treatment with this device or during an examination to confirm the effectiveness of the treatment, due to this device or examination, the person in charge of the research will take appropriate measures.

In addition, the company will purchase accident insurance (name of insurance company: Sompo Japan Insurance Inc.) for health damage caused by this equipment or examination (skin damage caused by the treatment equipment, fracture due to a fall during examination, trauma requiring hospital visit, etc.).

Amount of compensation (per person): Death and permanent disability 20 million yen

5,000 yen per day for hospitalization

3,000 yen per day for outpatient visits

15. Publication of Information on Clinical Research

This study will be registered in the database (Japan Registry of Clinical Trials) maintained by the Ministry of Health, Labour and Welfare prior to the implementation of the study. The registration will be updated as necessary according to changes in the research protocol and the progress of the study. When the study is completed, the results of the study will be promptly registered.

16. Duration of Clinical Research

April 1, 2021 - March 31, 2024 (expected date of recording the summary report summary in jRCT)

17. Explanation to and Consent of Subjects of Clinical Research

17-1. Procedures for obtaining informed consent, etc.

The principal investigator and subinvestigator(s) will provide the research subject with the consent explanation document approved by an accredited clinical research review committee, provide sufficient explanation in writing and orally, and obtain the subject's free and voluntary consent in writing.

When information that may affect the consent of research subjects is obtained, or when changes are made to the research plan that may affect the consent of research subjects, the principal

investigators and subinvestigators shall promptly provide information to research subjects and confirm in advance their willingness to participate in the research, and shall obtain prior approval from an approved clinical research review committee to revise the consent document and obtain their re-consent.

17-2. Procedures and Methods for Obtaining Informed Consent from a Surrogate, etc.

This study does not cover subjects who are unable to give informed consent due to communication due to dementia or other reasons, and does not anticipate that informed consent will be obtained from a surrogate or other person, so it is not applicable.

17-3. Procedures for obtaining informed assent

Not applicable.

17-4. Use and provision of samples and information for unspecified future research

There is no possibility that samples or information obtained from research subjects will be used for future research that is not identified at the time consent is obtained from the research subject or will be provided to other research institutions outside of the institution where this research is conducted and its affiliated institutions.

18. Other matters necessary for the proper conduct of clinical research

18-1. Conflicts of Interest Related to the Conduct of this Research

18-1-1. Summary

Research funding for this study was provided by the Longevity Medical Care Research and Development Fund and the Grants-in-Aid for Scientific Research for Basic Research. No funding is received from specific companies related to this research.。

18-1-2. Details of conflicts of interest with companies and other entities involved in this research project

1. 本 Research funding from companies manufacturing and marketing the drug under study, etc.: None
2. Provision or loan of drugs, equipment, or goods used in this research from companies or other entities related to this research at no charge or at a considerable discount: None
3. Services provided by companies, etc. related to this research: None
4. Persons employed by companies, etc. related to this research (including the past two years) engaged in this research: None

18-１-３. Details of Conflicts of Interest with Manufacturers and Distributors of Drugs, etc. Subject to this Research

We have confirmed that there are no conflicts of interest to be reported for all persons reporting conflicts of interest who are required to submit Conflict of Interest Management Form C.

18-2. Research in urgent and obvious life-threatening situations

Not applicable
