# Supplementary material for: Targeted vibratory therapy as a treatment for proprioceptive dysfunction: Clinical trial in older patients with chronic low back pain
Source: PLoS One. 2024 Jul 19;19(7):e0306898. doi: 10.1371/journal.pone.0306898 (PMC11259273; doi:10.1371/journal.pone.0306898)
Supplement: S2 File — (PDF) [file pone.0306898.s003.pdf]

研究計画書

＜研究課題名＞

固有感覚機能を基盤とした高齢者の腰痛に対する診断治療機器開発に関する研究

Diagnosis and Treatment Device Based on Proprioceptive Approach
for the Elderly Patients with Chronic Low Back pain

＜実施医療機関＞

国立研究開発法人　国立長寿医療研究センター

＜研究責任医師＞

国立研究開発法人　国立長寿医療研究センター

整形外科部長　酒井義人

２０２１ 年 ５ 月 １０ 日作成　　第２版

目次

1. 臨床研究の実施体制 ･･2
2. 臨床研究の背景 ･･4
3. 臨床研究の目的 ･･8
4. 臨床研究の内容 ･･9
5. 臨床研究の対象者の選択及び除外並びに中止に関する基準 ････････････11
6. 臨床研究の対象者に対する治療及び有効性・安全性の評価 ････････････12
7. 認定臨床研究審査委員会に対する疾病等報告 ･････････････････････15
8. 厚生労働大臣に対する疾病等報告 ･････････････････････････････16
9. 統計的な解析 ･･17
10. 原資料等の閲覧 ･･18
11. 品質管理及び品質保証 ･････････････････････････････････････18
12. 倫理的な配慮 ･･18
13. 記録（データを含む。）の取扱い及び保存 ･･･････････････････････20
14. 臨床研究の実施に係る金銭の支払及び補償 ･･･････････････････････20
15. 臨床研究に関する情報の公表 ････････････････････････････････20
16. 臨床研究の実施期間 ･･････････････････････････････････････21
17. 臨床研究の対象者に対する説明及びその同意 ･････････････････････21
18. その他臨床研究の適正な実施のために必要な事項 ･･････････････････21

略語及び用語の定義

略語又は用語一覧

略語又は用語	日本語又は英語
COP	足圧中心（Center of pressure）
RPW	固有受容加重比率（Relative Proprioceptive Weighting ratio
NRS	Numerical rating scale
RDW	Roland-Morris Disability Questionnaire
EQ5D	Euro QOL-5D
GDS	Geriatric depression scale
CSI	Central Sensitization Inventory

1. 臨床研究の実施体制

（1）研究形態

国立長寿医療研究センターのみで行われる研究

（2）研究者等

研究代表医師

国立長寿医療研究センター　整形外科部長　酒井義人

連絡先：

研究責任医師

国立長寿医療研究センター　整形外科部長　酒井義人

連絡先：

研究分担医師

国立長寿医療研究センター　整形外科部　整形外科医長　若尾典充

国立長寿医療研究センター　整形外科部　脊椎外科医長　松井寛樹

研究分担者

名古屋工業大学大学院　工学研究科電気・機械工学専攻　教授　森田良文

藤田医科大学　医療科学部臨床工学科　助教　山﨑一徳

研究協力者

愛知県三河青い鳥医療療育センター　三次元動作解析室　研究員　伊藤　忠

データマネジメントに関する責任者

所属）株式会社名古屋臨床薬理研究所　臨床研究事業部　部長　後藤倫代

連絡先：

統計解析に関する責任者

愛知県三河青い鳥医療療育センター　三次元動作解析室　研究員　伊藤　忠

連絡先：

モニタリングに関する責任者

（所属）株式会社名古屋臨床薬理研究所　伴　洋一郎

連絡先：

監査に関する責任者

なし

研究・開発計画支援担当者

国立長寿医療研究センター　整形外科　部長　酒井義人

連絡先：

調整管理実務担当者
（所属）株式会社名古屋臨床薬理研究所　臨床研究事業部　部長　後藤倫代
連絡先：

研究代表医師及び研究責任医師以外の研究を統括する者
名古屋工業大学大学院　工学研究科電気・機械工学専攻　教授　森田良文
連絡先：

（３）実施医療機関及び関連機関
国立長寿医療研究センター
住　所：愛知県大府市森岡町七丁目 430 番地
連絡先：

他の実施医療機関
なし

関連機関
名古屋工業大学
住所：愛知県名古屋市昭和区御器所町
担当者：工学研究科　教授　森田良文
業務内容：本研究に使用する診断治療機器の提供を行う

関連機関
藤田医科大学病院
住所：愛知県豊明市沓掛町田楽ケ窪１番地 98
担当者：医療科学部臨床工学科　助教　山崎一徳
業務内容：固有感覚機能の測定及び解析を行う

関連機関
愛知県三河青い鳥医療療育センター
住所：愛知県岡崎市高隆寺町小屋場９番地３
担当者：三次元動作解析室　研究員　伊藤　忠
業務内容：固有感覚機能の測定及び解析を行う

株式会社名古屋臨床薬理研究所（開発業務受託機関）
住所：愛知県名古屋市千種区吹上 1-2-15
担当者：代表取締役　伊藤順治
業務内容：症例登録システム及びデータベースの構築及び CSR の作成
監督方法：業務進捗について報告書を受けて確認を行う。

研究事務局
株式会社名古屋臨床薬理研究所
住所：愛知県名古屋市千種区吹上 1-2-15
担当者：代表取締役　伊藤順治

役割：研究を統括し、各種契約締結や資金管理等のマネジメントを担当する。

データセンター

株式会社名古屋臨床薬理研究所

住所：愛知県名古屋市千種区吹上 1-2-15

担当者：臨床研究事業部　部長　後藤倫代

役割：研究で得られたデータを収集し、試験全体の Quality Control を行う。

2. 臨床研究の背景

（1）国内外における対象疾患の状況

我が国では急速に高齢化が進んでおり、2015 年では 65 歳以上人口割合である高齢化率が 26.7%と過去最高の超高齢社会を迎え、今後もますます高齢化が進行すると言われている。国民有訴率については、平成 28 年調査において腰痛が男性で第一位、女性で第二位を占め、腰痛自体も加齢とともに増加する傾向があり、有訴者率の多いこの腰痛は高齢者の日常生活（ADL）を脅かし、ひいては要介護の要因となりうる。この腰痛の原因となる病態について近年、固有感覚受容(proprioception)と腰痛の関連の報告が散見される。固有感覚受容は姿勢制御にとって重要な感覚で、関節位置覚・運動覚・力学・筋収縮の大きさ・タイミングの知覚などを含む感覚であり、姿勢コントロールは腰痛患者で悪化しているとされる。海外の研究結果で、腰痛患者の中枢神経系は股関節や体幹からの proprioceptive なシグナルに頼るのではなく、下肢の入力シグナルにより頼っていることが示されている。（Brumagne S, et al. Proprioceptive weighting changes in persons with low back pain and elderly persons during upright standing. Neurosci Lett 2004; 366: 63-66.）研究者らは腰痛患者と非腰痛患者での比較対照試験においても、腰痛患者では下腿三頭筋でのバランス制御能に劣り、多裂筋優位の制御を行っていることを実験的に示した。(Ito T, Sakai Y, et al. Proprioceptive weighting ratio for balance control in static standing is reduced in elderly patients with non-specific low back pain. Spine 2018; 43(24):1704-1709.) また加齢に伴い固有感覚機能が低下することも同様の実験で示した。(Ito T, Sakai Y, et al. Postural strategy in elderly, middle-aged, and young people during local vibratory stimulation for proprioceptive inputs. Geriatrics 2018; 3(4): E93.）腰痛が高齢者に多い現状と、加齢に伴う固有感覚機能の低下から、固有感覚機能の低下は高齢者の腰痛の一因となり得ることは明らかである。

（2）これまでに実施されてきた標準治療の経緯及び内容

固有感覚機能の改善を目的とした治療としては、バランスボード、エクササイズボールなどの不安定な支持基底面上での立位や坐位でのエクササイズである固有感覚トレーニングが効果的であるとされる。しかし下肢筋力の低下した易転倒性を有する高齢者や転倒による骨折を容易に引き起こしうる骨粗鬆患者が多分に含まれる集団においては、このような機能訓練は危険性が有益性を上回ると考えられ、一般的には普及していない。

（3）現在の標準治療の内容及び治療成績

腰痛診療ガイドライン 2019（改訂第 2 版）によれば、慢性腰痛に効果的なエビデンスの強い治療法は薬物療法としてのセロトニン・ノルアドレナリン再取り込み阻害

剤（SNRI）、弱オピオイドのみで、中等度エビデンスを有する治療法として薬物療法としての非ステロイド性抗炎症薬（NSAIDs）、運動療法のみである。国内における全国的なランダム化試験の結果では、NSAIDs 内服と体幹筋強化・ストレッチによる運動療法の比較では、腰痛の強さには差はなく、腰痛関連の生活の質（QOL）の改善が運動療法で有意であった。しかし運動療法の種類についての明確な推奨はなく、また長期的な効果について、6 ヶ月までは有効であるが 1 年における長期において有用性は認められていない。また有害事象について、いずれも薬物も有害事象が懸念されるとされ、運動療法については有害事象について厳密に調査した報告はない。

（4）本研究の必要性につながる、現在の標準治療の課題及び不明点、並びに先行研究と比べた本研究の新規性及び独創性等

本研究は、医療機器の開発を目的とした臨床研究であり、固有感覚機能低下の正確な診断と、低下した固有感覚機能の向上およびそれに伴い腰痛の改善が得られるかを検証するものである。診断、治療いずれも先行研究と比較して新規性及び独創性を有する。

・診断について

固有感覚機能の評価として、体幹と下腿に 30-300Hz の周波数振動を連続的に与え、それらの振動刺激に対応する生体反応を閉眼起立した状態で重心動揺計により足圧中心の動揺を電気信号変化として測定する（図 1）ことで、機能低下している振動刺激に呼応する周波数帯を評価するものである。具体的には以下のように計測する。

<u>固有感覚機能の評価</u>

計測の手順は、振動刺激を腓腹筋と腰部多裂筋に交互に与え、合計 2 セット行う。計測条件は閉足、閉眼の両方を計測する。振動周波数は 30～300 Hz の周波数を自動的にスイープさせながら、ランダムに上昇パターンと下降パターンの 2 種類の振動刺激を与え平均を取る。1 回の測定時間は 75 秒であり、その間に 30～300 Hz の振動刺激が与えられる。残留振動の影響を排除する目的で各セット間に、60 秒間の座位休息を設ける（図 2）。振動子は伸縮性のゴムバンドで直接皮膚に固定することで試験中の振動部位の変化を防止する。振動刺激による前後方向の足圧中心（COP；Center of pressure）における平均位置の変化量から固有受容加重比率（Relative Proprioceptive Weighting ratio：RPW）を算出した。固有受容優位性に関する追加情報を得るために、相対的な固有受容加重比率 RPW を次の式により計算する。

$$RPW = \frac{(\text{abs dy GS})}{(\text{abs dy GS}) + (\text{abs dy LM})}$$

abs dy GS：下腿三頭筋刺激時の平均 COP の y 軸偏位

abs dy LM：腰部多裂筋刺激時の平均 COP の y 軸偏位

この計算式によって、腰部多裂筋と下腿三頭筋のどちらか、すなわち体幹か下肢かどちらを優位にして姿勢制御を行っているかを評価できる。RPW が 100%に近い程、腓腹筋の下肢優位の姿勢制御に相当し、0%に近い程、体幹優位の姿勢制御に相当する。

過去の固有感覚を評価する手法は、同様に振動刺激によるものであるが、筋紡錘を主

たる評価対象の固有感覚受容器としているため、60Hz 周囲の周波数に限定したものである。しかし、固有感覚機能を司る受容体には筋紡錘以外に、低周波数帯に呼応するマイスネル小体や高周波数帯に呼応するファーターパチニ小体、また同じ筋紡錘でも 60Hz 周囲以外にもより低周波数帯に呼応するものも存在する。本機器のうち診断部分における新規性として、単一周波数のみでなく連続的な広域周波数帯を網羅的に評価可能なことから、より詳細な固有感覚機能を評価しうることである。

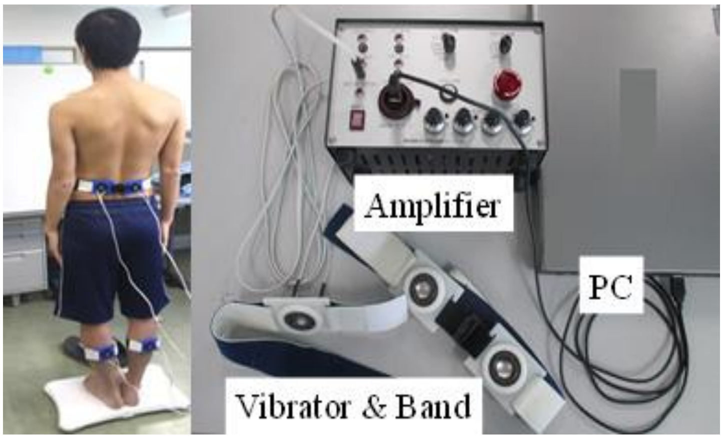

図 1. 周波数可変式加振器（右）と装着状態（左）

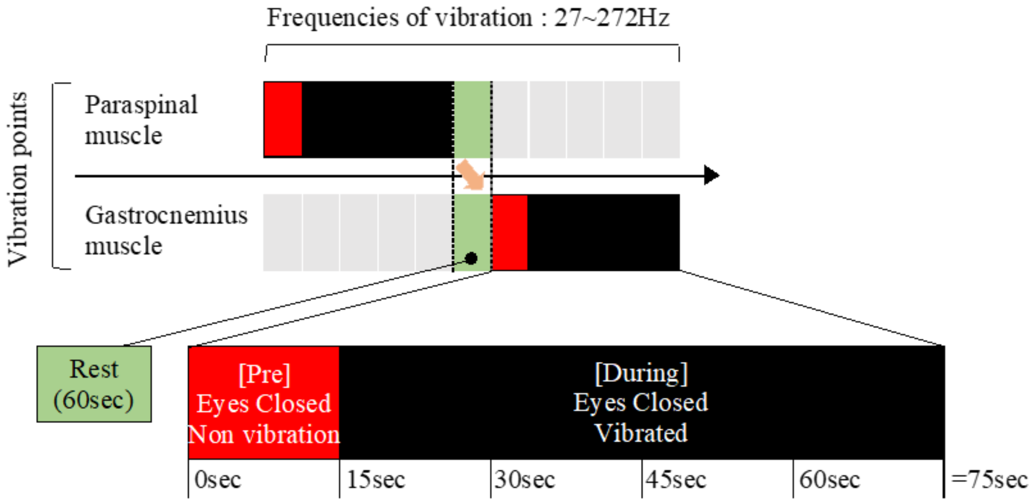

図 2. 診断手順

・治療について
振動刺激を利用した固有感覚機能低下の診断の先行研究は上述のごとく過去に報告があるが、その診断を基盤とした治療法についての研究報告はない。基礎研究で筋腱部に振動刺激を与えることで筋紡錘が活性化されることが報告されており、機能低下に陥っている特定周波数帯すなわち固有感覚受容器が判別できれば、当該周波数帯に振動刺激を付与することで機能低下している固有感覚受容器を活性化させることは理にかなっている。機能低下している周波数帯の診断そのものに新規性があり、この診断結果を基盤として機能低下している周波数帯に特定の当該周波数刺激を付与する治療法の開発に独創性を有する。

（5）本研究に用いる医薬品等に関する情報

1）当該医薬品等の名称（一般名及び販売名）

周波数可変式加振器

2）使用方法及び使用期間、並びにその設定根拠

研究実施医療機関において、上記（4）に記載した診断法により、体幹または下腿における固有感覚機能の低下の有無を確認し、機能低下していると考えられる周波数帯の振動刺激を機能低下している部位（体幹または下腿）に座位で１日３回（朝、昼、夕の食後）１分間、毎日 14 日間自宅で付与する。
なお、機器は「周波数可変式加振器　貸出管理表」を作成し医局にて管理する

<u>有害事象の検証</u>

上記(4)の方法で固有感覚機能の診断を行った後に、自宅治療のための周波数を設定し、当該周波数刺激を３分間の振動付与の後、２分間の休息を与え、再度上記（4）の方法で固有感覚機能を診断評価する。単回の振動付与により固有機能と腰痛が悪化しないか評価する。腰痛の評価は診断前および診断後の評価として、NRS を用いて行う。

<u>治療効果の検証</u>

上記（4）の方法で固有感覚機能の診断を実施医療機関で行い、機能低下部位および周波数帯を確定した後、ポータブル式加振器（図 3）を被験者に貸し出し、自宅において被験者自身で１回１分間、１日３回の振動付与を２週間施行してもらう。治療部位および周波数帯の設定は診断結果をもとに被験者に指示する。２週間後に実施医療機関において再度上記（4）の方法で固有機能の診断評価を行い、腰痛の評価を NRS および RDQ で行う。

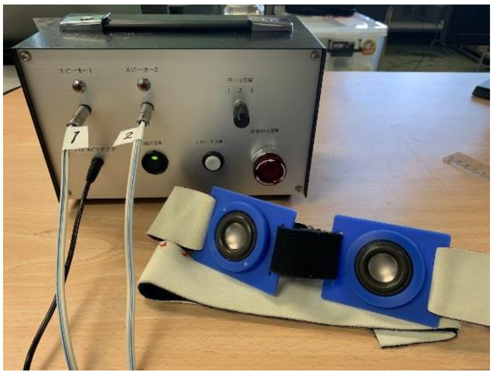

図３. ポータブル式加振器

<u>設定根拠</u>

・振動刺激時間：ボランティア 10 名を用いたパイロット試験ですべての周波数帯刺激を 15 分間行ったところ、１名において５分経過したところで腰に疲労感を訴えた。５分以上の持続的な振動付与は有害事象を誘発する可能性を考え、５分以下が望ましいと考えられた。先行研究で振動刺激に伴う固有感覚機能の回復は１分間で飽和するとの報告から、１回における治療的振動付与時間を１分、回数を１日３回（朝、昼、夕食後）と設定した。

（参考文献：Tjernström F et al. Adaptation of postural control to perturbations-a process that initiates long-term motor memory. Gait Posture. 2002 15:75-82, Capicikova N et al, Human posture response

to lower leg muscle vibration of different duration. Physiol Res. 2006 55; 129-134.）

・治療期間：慢性腰痛に対する弱オピオイド投与を行った研究実施機関の過去の試験において、有意な腰痛改善が得られたのが投与２週後であった。本薬物療法と同等の治療効果を期待することから、治療期間を２週間と設定した。

（参考文献：Sakai Y, et al. Pharmacological management of chronic low back pain in older patients: a randomized controlled trial of the effect of pregabalin and opioid administration. Eur Spine J. 2015 24(6) 1309-1317）

３）対象集団（年齢層、性別、疾患等）

65 歳以上の６ヶ月以上持続する腰痛を有し、薬物療法（NSAIDs、弱オピオイド、SNRI を含む）を１ヶ月以上投与により改善が得られない患者を対象とする。性別は問わず、胸腰椎における椎体骨折（骨粗鬆症性椎体骨折、外傷性骨折、病的骨折を含む）がないこととする。

４）当該医薬品等の有効性及び安全性に関して、非臨床試験、他の臨床研究等から得られている臨床的に重要な所見

本治療機器を用いた腰痛治療は非臨床試験、臨床研究とも前例がない。振動付与による固有感覚機能の改善については、筋紡錘が活性化することで得られるとの先行研究がいくつかある。（1976 Burke, 1989 Brumagne）ただし固有感覚機能の低下していない健常者に対して広範な周波数帯の振動刺激を付与することで、振動刺激の弊害として固有感覚機能が悪化することが報告されている。（2013 Blecher）本試験では、診断機器により固有感覚機能の診断を周波数帯別に行うことで、機能低下した固有感覚受容体のみに対して振動を付与することで安全に治療を行うことが可能である。

５）当該医療機器使用による利益及び不利益（既知のもの及び可能性のあるもの）

<u>診断における不利益</u>

本機器を用いて固有感覚機能を診断する際、閉眼起立を 60 秒間行う必要があるため、施行中の転倒リスクを伴う。そのため介助なくして起立姿勢が 60 秒間保持できない被験者は対象から除外する。また試験中は被験者の転倒に備え、転倒予防マットを配備した上で、必ず２名の男性補助員（研究責任医師または分担医師、研究分担者または研究協力者から２名）を同席させ転倒を予防する。診断機器開発において 200 例以上の高齢者での使用実績において転倒事故は１例も生じていない。

<u>治療における不利益</u>

持続的な振動による疼痛や疲労感の発生は５分以上付与しなければ生じないことを確認済みである。また、振動子の人体に触れる部位は耐水性があり破損する可能性は極めて低いと考えられる。万が一破損しても、漏電の危険性がなきよう、電極部には絶縁カバーが施してあり、安全性を保つ。漏電により人体に流れる電流値は 5mA 以下であり、人体に危険はなく、不快な状態も引き起こさない。

3. 臨床研究の目的

高齢者の身体的能力の低下の原因として、筋力・運動機能に加え固有受容感覚の低下が知られている。この固有感覚受容の低下を示す生体反応として、振動刺激に対する姿勢動揺が報告されており、この振動刺激に対する重心動揺を活用して、研究者らは低下した固有感覚機能を正確に診断するための機器を開発し、高齢者の固有感覚機

能低下が転倒や腰痛発生に関与し、加齢に伴う骨格筋量の減少（サルコペニア）の影響を受けていることを報告した。本研究では高齢者の固有感覚機能の改善を目的として、独自開発した診断装置の診断に従い適切な振動刺激を付与することで固有感覚機能の改善が得られ、固有感覚機能の改善が筋力や歩行機能、腰痛の改善に結びつくか検討することである。

4. 臨床研究の内容

4-1. 主要評価項目及び副次評価項目

（1）主要評価項目

機器による治療前（診断後）から治療 2 週間後の NRS スコアの変動

（2）副次評価項目

機器による診断前後の NRS スコアの変動および、機器による治療 2 週間後から治療終了 2 週間後の NRS スコアの変動

下記項目の機器による治療前から治療 2 週間後の変動、および治療 2 週間後の変動

・RDQ スコア
・Euro QOL-5D （EQ5D）
・Geriatric Depression Scale（GDS）
・Pain DETECT
・Central Sensitization Inventory（CSI）
・RPW 値

4-2. 研究デザイン

（1）介入

介入の有無：有り
介入の内容：腰痛患者における振動刺激の実施

（2）侵襲性

侵襲の有無：有り
侵襲の内容：体幹または下腿に対する振動刺激

（３）薬機法における未承認・適応外の医薬品等の臨床研究に該当するか

該当する（未承認医療機器使用）

（４）製薬企業等から資金提供を受けて実施される当該製薬企業等の医薬品等の臨床研究に該当するか

該当しない

（5）比較の方法

腰痛患者における振動刺激付与前後の比較

（6）無作為（ランダム化）割付

なし

（7）盲検性

非盲検（オープンラベル）

（8）臨床研究の手順

スクリーニングにより６ヶ月以上持続し薬物療法の効果が乏しい慢性腰痛である患者を選択し、研究同意を得る。同意取得後、データセンターへ登録用紙を送付し、症例登録を行う。同意取得より１ヶ月以内に固有感覚機能診断を行った後、機能低下した受容体に呼応する周波数振動刺激を設定し、機器を貸与し、２週間自宅治療を行う。治療開始後２週で、腰痛評価を行い治療終了とした後、さらに２週後（治療開始後より４週）に治療終了後の腰痛評価を再度行う。

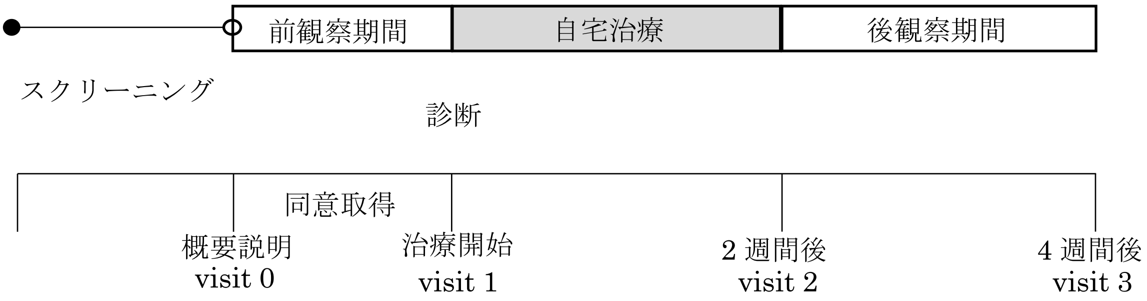

4-3. 使用する薬剤・機器及び管理について

ポータブル式加振器（製造販売承認未取得）

国立長寿医療研究センター　整形外科にて管理し被験者毎に貸し出す。

使用後は回収する。

4-4. 臨床研究の対象者の参加予定期間及び観察期間

同意取得から診断（visit １）まで１ヶ月以内

治療開始から治療終了まで２週間（治療開始と治療終了時にデータ収集）

治療終了から観察期間２週間（治療終了２週後にデータ収集）

4-5. 臨床研究の中止基準

以下のような場合は臨床研究を中止する。

1. 患者に予期できない、又は許容できない重要なリスクがあると判断した場合
2. 患者あるいは代諾者より同意の撤回があった場合
3. 患者あるいは代諾者より治療の変更・中止の申し出があった場合
4. 有害事象の発現（原疾患の増悪、合併症の悪化、新たな疾患の併発等）により、主任研究者または研究補助者が試験の継続を不適当と判断した場合
5. 転居等により決められた来院日に通院出来なくなった場合
6. 本試験に起因しない原因により死亡または重篤な QOL 悪化を招き、評価継続が不能となったもの

7. その他、主任研究者が試験の継続を不適当と判断した場合

特定臨床研究を中止すると判断した場合は、中止から 10 日以内に承認を得た認定臨床研究審査委員会に通知し、厚生労働大臣に届け出る。

4-6. 症例報告書に直接記入され、かつ原資料と解すべき内容の特定

- 診療記録（検査記録、投薬内容、単純エックス線写真、MRI 画像、DXA 画像）
- Numerical Pain Rating Scale （NRS）調査用紙
- Roland-Morris Disability Questionnaire（RDQ）調査用紙
- Euro QOL-5D（EQ5D）調査用紙
- Geriatric Depression Scale（GDS）調査用紙
- Pain DETECT 調査用紙
- Central Sensitization Inventory（CSI）調査用紙
- 治療日誌
- 固有感覚機能の評価結果

5. 臨床研究の対象者の選択及び除外並びに中止に関する基準

5-1. 選択基準

1. 本研究の参加にあたり、十分な説明を受けた後に、十分な理解の上に、本人の自由意志による文書同意が得られた患者。
2. 同意取得時に 65 歳以上の患者。
3. 6 ヶ月以上持続し薬物療法で改善が得られない NRS 値 3 以上の慢性腰痛を有する患者。
4. 研究開始より 3 ヶ月以内に施行された腰椎エックス線、腰椎 MRI 等で、新鮮椎体骨折を認めない患者。
5. Visit 1 における固有感覚機能診断において、Z score＜-1.0 を基準とした固有感覚機能低下を認めるもの。

（設定根拠）

1. 十分な理解の上、自らの自由意志を示せる患者を対象とするため。
2. 先行研究で 65 歳以上の高齢者において有意な固有感覚機能低下が認められたため。
3. 自然経過で改善されない、一定以上の強さの腰痛を治療対象としているため。（NRS 値 3 未満では疼痛が少ないと考えられている）
4. 椎体骨折の治癒過程で腰痛の自然軽快が考えられるため。
5. 固有感覚機能が低下した腰痛患者を研究対象とするため。

5-2. 除外基準

1. 疼痛、麻痺等の運動機能低下や栄養障害により起立保持に介助を要するもの、介助なしで 60 秒間起立姿勢が保持できないもの
2. 脳梗塞、脳出血、脳・脊髄腫瘍、頚髄症・胸髄症などの中枢神経疾患、筋ジストロフィーなどの筋疾患を伴うもの
3. 骨粗鬆症の有無を問わず、同意所得より 1 年以内に椎体骨折の既往のある

もの
4. 非ステロイド性抗炎症薬（NSAIDs）を定期的に服用しているもの
5. 認知症、意識障害などにより意思疎通の困難なもの
6. 研究期間中に治療を要する疾患を有するもの
7. 高齢者の転倒リスク簡易評価（鳥羽）で６点以上のもの
8. 精神疾患で精神科または心療内科通院中のもの
9. 本研究に対する同意が得られないもの
10.その他、研究責任医師、研究分担医師が不適切と判断した患者

（設定根拠）
1. 治療開始前の固有感覚機能の診断が十分に施行できないため。
2. 治療開始前の固有感覚機能の診断が十分に施行できないため。
3. 椎体骨折の治癒過程で腰痛の自然軽快が考えられるため。
4. 強い腰痛を有する場合は検査中に痛みが悪化する恐れがあるため
5. 適切な腰痛評価ができない可能性があるため。
6. 研究期間中の疾患治療介入による影響が懸念されるため。
7. 検査中の転倒の恐れがあるため。
8. 心因性の腰痛が否定できないため。
9. 研究対象者の安全性確保のために設定した。
10.研究対象者の安全性確保のために設定した。

5-3. 臨床研究の対象者ごとの中止基準

研究責任医師及び研究分担医師が、次に上げる理由等で機器による治療継続が不可能と判断した場合には、当該研究対象者への機器による治療は中止する。その際、研究対象者に中止する旨及びその理由を説明し、必要な治療・観察を実施する。
1. 有害事象の発現により機器による治療継続が好ましくないと判断された場合
2. 機器による治療後に研究対象者として不適格であることが判明した場合
3. 臨床研究の対象者が臨床研究参加の同意を撤回した場合

（設定根拠）
1～2. 臨床研究の対象者の安全性を確保するために設定した。
１については、腰痛悪化のため非ステロイド性抗炎症薬（NSAIDs）の服用を追加した場合も含む。
3. 臨床研究の対象者の倫理性を確保するために設定した。

6. 臨床研究の対象者に対する治療及び有効性・安全性の評価

6-1. 治療スケジュール（表 1）

機器による治療期間

固有感覚機能診断により決定された治療部位に設定された周波数の振動刺激を、１日３回（朝、昼、夕食後）、１回１分

観察期間

機器による治療期間後２週間

来院スケジュール

同意取得後の登録・固有感覚機能診断日（治療開始前日）、機器による治療開始２週後、観察期間終了時（治療開始４週後）

食事制限　　なし

表 1. 来院および治療スケジュール

	Visit 0	Visit 1		Visit 2		Visit 3
		Day0	Day1 ～13	Day14	Day15 ～27	Day28
		診断•評価	自宅治療	評価	後観察期間	
来院	●	●		●		●
同意取得	●					
症例登録	●					
患者背景	●					
身長・体重		●				
併用薬の確認	●	●		●		●
体温		●		●		●
血圧		●		●		●
皮膚所見	●	●		●		●
起立、歩行状態の確認	●	●		●		●
腰痛評価	●	●		●		●
Numerical rating scale (NRS)		●*		●		●
RDQ スコア		●		●		●
EuroQOL-5D (EQ5D)		●		●		●
Geriatric Depression Scale（GDS）		●		●		●
Pain DETECT		●		●		●
Central Sensitization Inventory（CSI）		●		●		●
固有感覚機能診断: RPW 値		●		●		●
周波数振動刺激の設定		●				
周波数可変式加振器治療**		●	●	●		
治療持続効果の検証（RPW 値）				●		●

*visit 1 における NRS 評価は、機器による診断前後の 2 回施行する。
**加振器治療は 1 日 3 回（朝、昼、夕食後）、計 42 回行う。visit1 を夕に設定し、visit1 における診断後の振動付与を初回治療とし、自宅治療における Day14 の昼の治療を最終とする。

Numerical Rating Scale （NRS）：腰痛の強さの評価
Roland-Morris Disability Questionnaire（RDQ）：腰痛による ADL 障害の評価
Euro QOL-5D（EQ5D）：高齢者の QOL 評価
Geriatric Depression Scale（GDS）：うつ傾向の評価
Pain DETECT：神経障害性疼痛の評価
Central Sensitization Inventory（CSI）：中枢性感作の評価

6-2. 併用制限（禁止）薬・併用制限（禁止）療法

併用制限（禁止）薬：特になし、ただし同意取得から観察期間終了まで腰痛またはそれ以外の疼痛に対する新規薬物治療の開始、および同意取得前の治療薬の変更は行わないこと。

併用制限（禁止）療法： 特になし、ただし同意取得から観察期間終了まで腰痛またはそれ以外の疼痛に対する新規療法（運動療法、理学療法など）の開始、および同意取得前の療法（運動療法、理学療法など）の変更は行わないこと。

6-3. その他の取り決め事項

なし

6-4. 有効性の評価

（1） 主要評価項目

機器による治療前から治療 2 週間後の NRS スコアの変動

（2）副次評価項目

機器による診断前後の NRS スコアの変動および、機器による治療 2 週間後から治療終了 2 週間後の NRS スコアの変動

下記項目の機器による治療前から治療 2 週間後の変動、および治療 2 週間後から治療終了 2 週間後の変動

・RDQ スコア
・Euro QOL-5D （EQ5D）
・Geriatric Depression Scale（GDS）
・Pain DETECT
・Central Sensitization Inventory（CSI）
・RPW 値

NRS は腰痛の強さを示す評価指標である。固有感覚診断時（visit 1）、機器による治療 2 週後（visit 2）、治療終了 2 週後（visit 3）に実施する。

RDQ スコアは腰痛による ADL 評価指標である。固有感覚診断時（visit 1）、機器による治療 2 週後（visit 2）、治療終了 2 週後（visit 3）に実施する。

Euro QOL-5D （EQ5D）は高齢者の QOL 評価指標である。固有感覚診断時（visit 1）、機器による治療 2 週後（visit 2）、治療終了 2 週後（visit 3）に実施する。

Geriatric Depression Scale（GDS）はうつの評価指標である。固有感覚診断時（visit 1）、機器による治療 2 週後（visit 2）、治療終了 2 週後（visit 3）に実施する。

Pain DETECT は神経障害性疼痛の評価指標である。固有感覚診断時（visit 1）、機器による治療 2 週後（visit 2）、治療終了 2 週後（visit 3）に実施する。

Central Sensitization Inventory（CSI）は中枢性感作の評価指標である。固有感覚診断時（visit 1）、機器による治療 2 週後（visit 2）、治療終了 2 週後（visit 3）に実施する。

RPW 値は固有感覚機能の評価指標である。固有感覚診断時（visit 1）、機

器による治療２週後（visit ２）、治療終了２週後（visit ３）に実施する。

解析の方法は「9. 統計的な解析」を参照のこと。

6-5. 安全性の評価

（1）身体所見

皮膚の状態（かゆみ、発赤、不快感など）の確認

（2）バイタルサイン

血圧、体温測定

（3）腰痛の評価

機器使用中の腰痛の悪化

身体所見、バイタルサインの確認は固有機能診断時および治療２週後、治療終了２週後（visit 1, 2, 3）の際の検査前に評価する。

体温 37.5℃以上、収縮期血圧 180 以上の場合は検査を中止する。また、機器を用いた診断、治療中に腰痛が悪化した場合も中止とする。機器による治療開始にあたり、固有感覚診断時（visit １）に診断後に刺激周波数を決定した後に治療としての初回振動刺激を試験的に付与し、１ 回の刺激前後での腰痛 NRS が悪化していたものは本研究への参加を中止する。機器による治療開始以後に疾病並びに検査中止事案が発生した場合は、研究責任医師の診察により適切な診療科受診へ誘導するとともに、腰痛の悪化・皮膚疾患については発生後２週間、その他、未知で研究の実施との関連が疑われる疾病については研究終了又は症状の改善が確認されるまで経過観察を行う。

7. 認定臨床研究審査委員会に対する疾病等報告

次に掲げる事項を知ったときは、研究責任医師はそれぞれに定める期間内に実施医療機関の管理者に報告した上で、研究代表医師に通知し、その旨を速やかに各施設の研究責任医師に情報提供する。

各施設の研究責任医師は速やかに当該情報提供の内容を実施医療機関の管理者に報告する。

研究代表医師は、実施医療機関の管理者に報告した上で、認定臨床研究審査委員会に報告する。

ア　以下の疾病等の発生のうち、未承認又は適応外の医薬品等を用いる特定臨床研究の実施によるものと疑われるものであって予測できないもの

７日

一　死亡

二　死亡につながるおそれのある疾病等

イ　以下の疾病等の発生のうち、未承認又は適応外の医薬品等を用いる特定臨床研究の実施によるものと疑われるもの（アに掲げるものを除く。）

15 日

一　死亡

二　死亡につながるおそれのある疾病等

ウ　以下の疾病等の発生のうち、未承認又は適応外の医薬品等を用いる特定臨床研究の実施によるものと疑われるものであって予測できないもの（アを除く。）

15 日

一　治療のために医療機関への入院又は入院期間の延長が必要とされる疾病等
二　障害
三　障害につながるおそれのある疾病等
四　一から三まで並びに死亡及び死亡につながるおそれのある疾病等に準じて重篤である疾病等
五　後世代における先天性の疾病又は異常

なお、特定臨床研究の実施に起因するものと疑われる疾病等（上記のものを除いた、既知で非重篤な事象）は認定臨床研究審査委員会への定期報告を行うときに報告する。

また、研究責任医師は、当該特定臨床研究に用いる医療機器（又は再生医療等製品）の不具合の発生であって、当該不具合によって以下の疾病等が発生するおそれのあるものについて知ったときは、これを知った日から 30 日以内にその旨を実施医療機関の管理者に報告した上で、研究代表医師に通知し、その旨を速やかに各施設の研究責任医師に情報提供する。

各施設の研究責任医師は速やかに当該情報提供の内容を実施医療機関の管理者に報告する。

研究代表医師は、実施医療機関の管理者に報告した上で、認定臨床研究審査委員会に報告する。

ア　死亡
イ　死亡につながるおそれのある疾患等
ウ　治療のために医療機関への入院又は入院期間の延長が必要とされる疾病等
エ　障害
オ　障害につながるおそれのある疾病等
カ　ウからオまで並びに死亡及び死亡につながるおそれのある疾病等に準じて重篤である疾病等
キ　後世代における先天性の疾病又は異常

8. 厚生労働大臣に対する疾病等報告

研究責任医師は、次に掲げる事項を知ったときは、それぞれに定める期間内に厚生労働大臣に報告する。

ア　以下の疾病等の発生のうち、未承認又は適応外の医薬品等を用いる特定臨床研究の実施によるものと疑われるものであって予測できないもの
7日
一　死亡
二　死亡につながるおそれのある疾病等

イ　以下の疾病等の発生のうち、未承認又は適応外の医薬品等を用いる特定臨床研究の実施によるものと疑われるものであって予測できないもの
15日
一　治療のために医療機関への入院又は入院期間の延長が必要とされる疾病等
二　障害

三　障害につながるおそれのある疾病等
四　一から三まで並びに死亡及び死亡につながるおそれのある疾病等に準じて重篤である疾病等
五　後世代における先天性の疾病又は異常

9. 統計的な解析

9-1. 解析対象集団

最大の解析対象集団（Full Analysis Set: FAS）は機器による治療を 1 回以上行い、その後も何らかの観察が行われた全ての被験者とする。ただし適格性基準に該当しない被験者は除外する。

実施計画書に適合した対象集団（Per Protocol Set: PPS）は FAS のうち、実施計画書の規定から重大な逸脱がなく、主要評価項目の評価が可能な被験者とする。

安全性解析対象集団は機器による治療を 1 回以上施行された全ての被験者とする。

9-2. 有効性における統計解析手法

主要評価項目である NRS スコアの 2 週後の値は治療開始前（診断後）の値との比較において、対応のある 2 群の平均値差の検定を用いて解析する。主要な解析は FAS を用いて行う。FAS における治療前および 2 週後の NRS スコアがともに正規性を有さない場合は、Wilcoxon の符号付き順位和検定を行う。また、治療終了後の治療維持効果を評価する目的で、4 週後の値を 2 週後の値との比較対応のある 2 群の平均値差の検定を用いて解析する。4 週後および 2 週後の NRS スコアのどちらか一方が正規性を有さない場合は、Wilcoxon の符号付き順位和検定を行う。

副次的評価項目である RDQ、EQ5D、GDS、Pain DETECT、CSI の各スコアおよび RPW 値は PPS を用いて行う。2 週後の値を治療開始前の値との比較対応のある 2 群の平均値差の検定を用いて解析するが、PPS における各スコアのどちらか一方が正規性を有さない場合は、Wilcoxon の符号付き順位和検定を行う。また、治療終了後の治療維持効果を評価する目的で、4 週後の値を 2 週後の値との比較対応のある 2 群の平均値差の検定を用いて解析する。4 週後および 2 週後の副次評価項目の各スコアのどちらか一方が正規性を有さない場合は、Wilcoxon の符号付き順位和検定を行う。

9-3. 中間解析における統計解析手法

中間解析は行わず、統計解析は研究終了後に 9-2 の手法で行う。

9-4. 予定解析症例数及びその根拠

予定解析症例

試験全体　30 例

（予定症例の設定根拠）

高齢者（65 歳以上）の慢性腰痛患者における弱オピオイド投与試験（N=30）における腰痛 NRS のデータを使用し、本薬物治療と同等の改善効果が得ら

れることを仮説とした。治療前後（2 週間）の NRS 平均値の差 1.7、共通の標準偏差 3.1、αエラー0.05、検出力 0.08 を “Easy-R”（EZR）(Saitama Medical Center, Jichi Medical University, Saitama, Japan)を用いて対応のある 2 群の平均値の比較のためのサンプルサイズ計算で、必要サンプルサイズ N=29、検出力 0.813 と算出されたため、予定症例数を 30 例と設定した。

（参考文献：Sakai Y, et al. Pharmacological management of chronic low back pain in older patients: a randomized controlled trial of the effect of pregabalin and opioid administration. Eur Spine J. 2015 24(6) 1309-1317）

9-5. 臨床研究の中止基準

5.5　臨床研究の中止基準 参照

9-6. 欠落、不採用及び異常データの取扱いの手順

2 週および 4 週時点での NRS スコアの欠損は不採用とする。
治療日誌より、施行回数が 50%以下であったものは不採用とする。

9-7. 当初の統計的な解析計画を変更する場合の手順

当初の統計的な解析計に変更が生じた場合は、研究計画書を改訂し、臨床研究の統括報告書において記載する。

10. 原資料等の閲覧

研究責任医師は、臨床研究に関連するモニタリング、認定臨床研究審査委員会及び国内外規制当局の調査の際に、原資料等（資金提供者との契約の内容を含む。）の全ての臨床研究関連記録について、直接閲覧に協力する。

11. 品質管理及び品質保証

11-1. モニタリングの方法

モニタリング手順書に準じて、モニタリングを行う。

12. 倫理的な配慮

12-1. 研究対象者の負担、予測されるリスク

本研究に参加することで以下の危険が予測される。

・転倒

固有感覚機能評価には閉眼で起立した状態で行う検査が含まれるため、検査中に転倒する危険がある。

・皮膚障害

治療機器を 5 分間腰背部または下腿後面に当てて振動刺激を与えるため、振動子の接触による皮膚障害として発赤、かゆみを生じる可能性がある。

・機器の破損および漏電

振動子の人体に触れる部分は耐水性があり、使用中に破損する可能性は極めて低い。万一破損した場合でも漏電の危険がなきよう電極部には絶縁カバーを施してあり、安全性を担保してある。漏電による人体に流れる電流値は 5mA 以下であり人体に危険が及ぶ可能性はなく、また不快な

感覚も生じないと予測される。
また本研究に参加することで以下の負担の発生が考えられる。
・精神的苦痛
本研究に協力しなければならないと思われることで発生する可能性がある。その際は直ちに研究参加を中止することが可能であることを事前に十分に説明する。
・評価のための通院
　固有感覚診断時（visit 1）、機器による治療２週後（visit 2）、治療終了２週後（visit 3）の計３度の通院を要する。時間的および交通手段使用による経済的負担を考慮し、負担軽減費として 1 回通院あたり 2,000 円を支払う。（振り込み又はクオカードでの支払いを予定）

12-２. 予測される利益
　本研究へ参加することにより、腰痛の改善が得られることで日常生活動作（ADL）および生活の質（QOL）の向上が期待される。また固有感覚機能の向上により歩行のしやすさ、易転倒性の減少が期待できる。研究対象者への直接的な利益が生じなかった場合においても、研究成果により将来の医療の進歩に貢献できる可能性がある。

12-3. 負担、リスク、利益の総合評価
・転倒
検査中の被験者の転倒について、スクリーニングにおいて医師による転倒リスク評価を高齢者の転倒リスク簡易評価（鳥羽）を行い６点以上の重度の易転倒性を有すると考えられるものは基準に入れない。また軽度の易転倒性を有するものであっても、本機器による治療で固有感覚機能の改善される可能性があることから、易転倒性に対する改善効果も期待できることを説明する。
・皮膚障害
スクリーニングにおいて皮膚疾患、アレルギー、外用剤等によるかぶれやすさなどについての問診を行う。起こりうる皮膚障害については一時的であり、検査および治療の中断により改善すると考えられること、苦痛と感じた場合には自らの意思で直ちに研究を中断することが可能であることを説明する。
・機器の破損および漏電
機器の破損に関してはよほどの外的衝撃を与えなければ起こらないことを説明し、万が一漏電しても人体に危害が及ぶ電流は発生しないことを説明する。
上記のリスクが生じる可能性を勘案しても、本機器による治療により腰痛が改善されるというメリットが生じうることを説明する。
また精神的負担、通院による負担については、被験者選択において腰痛治療で整形外科通院中の患者を対象にしていることから、主治医としての研究責任医師または分担医師との間で信頼関係が得られていることを想定している。被験者としても腰痛改善のための意欲があるものを対象としているため、実施内容を勘案しても精神的負担は大きくならないと想定する。
また被験者に居住地を考慮し、通院のための経済的負担をなくすことで実

質的な負担軽減になるものと考える。

12-4.負担・リスクを最小化する対策

転倒に対する予防として、起立時の検査中は機器操作者を除いて測定に携わる研究者または補助員を必ず 2 名以上同席させ、特に閉眼時の転倒に備え患者を支える。万が一の転倒でも健康被害が発生しないよう、測定器の周辺には転倒予防マットを床に置く。測定中あるいは測定後に疲労感を感じる可能性があるため、十分に休息をとって頂くことや、十分な水分補給をして頂くなどの対処を行い、被検者より研究継続困難である意思表示があった場合は、その時点で速やかに試験終了とする。

13. 記録（データを含む。）の取扱い及び保存

本研究で取得する以下の資料は研究責任医師の責任のもと、データはパスワードをかけ、また紙資料は鍵のかかる棚に厳重に保管する。保管期間は当該特定臨床研究が終了した日から５年間とする。破棄する際は復元不可能な形（シュレッダー処理や焼却処分等）で処理する。

1）研究計画書、実施計画、特定臨床研究の対象者に対する説明及びその同意に係る文書、総括報告書その他のこの省令の規定により研究責任医師が作成した文書又はその写し
2）認定臨床研究審査委員会から受け取った審査意見業務に係る文書
3）モニタリングに関する資料
4）原資料等
5）特定臨床研究の実施に係る契約書
6）特定臨床研究に用いる医薬品等の概要を記載した文書

また本研究で取得した試料・情報は、個人が特定できない形（被験者番号）で管理され、統計解析責任者の所属施設である愛知県三河青い鳥医療療育センターに提供し、解析を進める。提供を受けたデータに関しては、提供先においてデータはパスワードをかけ、また紙資料は鍵のかかる棚に厳重に保管する。保管期間は当該特定臨床研究が終了した日から５年間とする。破棄する際は復元不可能な形（シュレッダー処理や焼却処分等）で処理する。

14. 臨床研究の実施に係る金銭の支払及び補償

本研究の実施に伴い、本機器による治療中および治療効果確認のための検査中において研究対象者に健康被害が本治療機器または検査に起因して発生した場合は、研究担当者は適切な処置を講じる。

また本機器または検査に起因する健康被害（治療機器による皮膚障害、検査中の転倒による骨折および通院を要する外傷等）については、傷害保険（保険会社名：損害保険ジャパン株式会社）に加入する。

補償金額（一人につき）：死亡・後遺障害　2,000 万円
入院日額　5,000 円
通院日額　3,000 円

15. 臨床研究に関する情報の公表

本研究は、研究の実施に先立って厚生労働省が整備するデータベース（Japan Registry of Clinical Trials）に登録する。また、研究計画書の変更及び研究の進

捗に応じて適宜登録内容を更新する。研究が終了したときは、すみやかに本研究の結果を登録する。

16. 臨床研究の実施期間

2021 年 4 月 1 日～2024 年 3 月 31 日（総括報告書の概要を jRCT に記録する予定日）

17. 臨床研究の対象者に対する説明及びその同意

17-1. インフォームド・コンセントを得る手続き等

研究責任医師及び研究分担医師は、認定臨床研究審査委員会で承認の得られた同意説明文書を研究対象者に渡し、文書及び口頭による十分な説明を行い、研究対象者の自由意思による同意を文書で取得する。

研究責任医師及び研究分担医師は、研究対象者の同意に影響を及ぼす情報が得られたときや、研究対象者の同意に影響を及ぼすような実施計画等の変更が行われるときは、速やかに研究対象者に情報提供し、研究に参加するか否かについて研究対象者の意思を予め確認するとともに、事前に認定臨床研究審査委員会の承認を得て同意説明文書等の改訂を行い、研究対象者の再同意を得ることとする。

17-2. 代諾者等からインフォームド・コンセントを受ける場合の手続及び方法

本研究では対象者本人が認知症等により意思疎通で、インフォームド・コンセントを与えることができないものは対象としておらず、代諾者等からインフォームド・コンセントを受けることは想定していないため、該当しない。

17-3. インフォームド・アセントを得る場合の手続

該当しない。

17-4. 特定されない将来の研究のための試料・情報の使用・提供

研究対象者から取得された試料・情報について、研究対象者から同意を受ける時点では特定されない将来の研究のために用いられる可能性又は本研究実施機関及び関連機関以外の他の研究機関に提供される可能性はない。

18. その他臨床研究の適正な実施のために必要な事項

18-1. 本研究実施に係る利益相反

18-1-1. 概要

この研究の研究資金は長寿医療研究開発費、科学研究費助成事業基盤研究である。本研究に関連のある特定の企業からの資金提供は受けない。

18-１-２. 本研究課題と関わりのある企業等との利益相反の内容

1. 本研究対象医薬品等製造販売企業等からの研究資金の提供：なし
2. 本研究に関連する企業等からの本研究に使用する薬剤・機器・物品等の、無償あるいは相当程度安価での提供・借用：なし
3. 本研究に関連する企業等からの役務提供：なし
4. 本研究に関連する企業等在籍者（過去２年を含む）の本研究への従事：なし

18-1-3．本研究対象医薬品等の製造販売業者等との利益相反の内容
利益相反管理様式Ｃの提出が必要な全ての利益相反申告者について、申告すべき利益相反はないことを確認した。

18-2. 緊急かつ明白な生命の危機が生じている状況における研究
該当なし
