# Supplementary material for: Targeted vibratory therapy as a treatment for proprioceptive dysfunction: Clinical trial in older patients with chronic low back pain
Source: PLoS One. 2024 Jul 19;19(7):e0306898. doi: 10.1371/journal.pone.0306898 (PMC11259273; doi:10.1371/journal.pone.0306898)
Supplement: Attachment — Submitted filename: March 6 2024-PONE-D-23-38713.pdf [file pone.0306898.s004.pdf]

PLOS ONE

Targeted vibratory therapy as a treatment for proprioceptive dysfunction: clinical trial in older patients with chronic low back pain

--Manuscript Draft--

**Manuscript Number:**	PONE-D-23-38713
**Article Type:**	Clinical Trial
**Full Title:**	Targeted vibratory therapy as a treatment for proprioceptive dysfunction: clinical trial in older patients with chronic low back pain
**Short Title:**	Targeted vibratory therapy for proprioceptive dysfunction
**Corresponding Author:**	Yoshihito Sakai, MD, PhD<br>National Center for Geriatrics and Gerontology<br>Obu, None Selected JAPAN
**Keywords:**	chronic low back pain; vibratory therapy; proprioception
**Abstract:**	Background<br>Proprioceptive function declines with age, leading to falls, pain, and difficulties engaging in activities of daily living among older adults. Although individuals with low back pain (LBP) have decreased lumbosacral proprioception in various postures, the mechanism by which reduced proprioceptive function causes LBP remains uncertain. Vibratory stimulation may enhance proprioceptive function; however, its efficacy as a treatment for LBP has not been investigated. This study investigated the feasibility of improving proprioceptive function and its effect on alleviating chronic LBP in older patients through targeted vibratory therapy (TVT) administration.<br>Methods<br>Older patients aged >65 years with nonspecific chronic LBP were recruited. TVT was performed by applying vibratory stimulation corresponding to the frequency of dysfunctional receptors for 1 min for 14 days to activate proprioceptors, with patients performing TVT three times daily at home. In cases with reduced proprioceptive function at multiple sites, the targeted treatment was aimed at the lowest frequency band value. Biological responses to vibratory stimulation targeting reduced proprioception in the trunk or lower legs were measured at sites with impaired function.<br>Findings<br>Overall, 56 patients with chronic LBP were enrolled, of whom 32 were recruited for treatment based on a proprioceptive dysfunction diagnosis. No adverse events were observed in any patient. After 2 weeks of TVT, numerical pain rating scale score improved to <3 in 78.1% of patients, 73.1% achieved a score ≤ 3, proprioceptive function improved in 81.3% in cases, and engagement in activities of daily living improved significantly.<br>Conclusion<br>TVT, involving vibratory stimulation at a receptor-specific frequency, demonstrated efficacy in improving proprioception and alleviating LBP in older patients with impaired proprioceptive function without affecting non-targeted proprioceptors.
**Order of Authors:**	Yoshihito Sakai, MD, PhD
	Yoshifumi Morita, PhD
	Keitaro Kawai, MEng
	Jo Fukuhara, BEng
	Tadashi Ito, PhD
	Tsuyoshi Watanabe, PhD
	Norimitsu Wakao, PhD
	Hiroki Matsui, PhD
**Additional Information:**	
**Question**	**Response**

Powered by Editorial Manager® and ProduXion Manager® from Aries Systems Corporation

**Financial Disclosure**<br><br>Enter a financial disclosure statement that describes the sources of funding for the work included in this submission. Review the submission guidelines for detailed requirements. View published research articles from *PLOS ONE* for specific examples.<br><br>This statement is required for submission and **will appear in the published article** if the submission is accepted. Please make sure it is accurate.<br><br>**Funded studies**<br>Enter a statement with the following details:<br>• Initials of the authors who received each award<br>• Grant numbers awarded to each author<br>• The full name of each funder<br>• URL of each funder website<br>• Did the sponsors or funders play any role in the study design, data collection and analysis, decision to publish, or preparation of the manuscript?<br><br>Did you receive funding for this work?	The author(s) received no specific funding for this work.
**Competing Interests**<br><br>Use the instructions below to enter a competing interest statement for this submission. On behalf of all authors, disclose any competing interests that could be perceived to bias this work—acknowledging all financial support and any other relevant financial or non-financial competing interests.<br><br>This statement is required for submission and **will appear in the published article** if the submission is accepted. Please make sure it is accurate and that any funding sources listed in your Funding Information later in the submission form are also declared in your Financial Disclosure statement.	no competing interests exist

View published research articles from *PLOS ONE* for specific examples.<br><br>**NO authors have competing interests**<br><br>Enter: *The authors have declared that no competing interests exist.*<br><br>**Authors with competing interests**<br><br>Enter competing interest details beginning with this statement:<br><br>*I have read the journal's policy and the authors of this manuscript have the following competing interests: [insert competing interests here]*<br><br>* typeset	
**Ethics Statement**<br><br>Enter an ethics statement for this submission. This statement is required if the study involved:<br><br>• Human participants<br>• Human specimens or tissue<br>• Vertebrate animals or cephalopods<br>• Vertebrate embryos or tissues<br>• Field research<br><br>Write "N/A" if the submission does not require an ethics statement.<br><br>General guidance is provided below. Consult the submission guidelines for detailed instructions. **Make sure that all information entered here is included in the Methods section of the manuscript.**	the University of Fujita Health University Certified Review Board (CRB4180003)<br>All participants provided written informed consent.

**Format for specific study types**<br><br>**Human Subject Research (involving human participants and/or tissue)**<br>• Give the name of the institutional review board or ethics committee that approved the study<br>• Include the approval number and/or a statement indicating approval of this research<br>• Indicate the form of consent obtained (written/oral) or the reason that consent was not obtained (e.g. the data were analyzed anonymously)<br><br>**Animal Research (involving vertebrate animals, embryos or tissues)**<br>• Provide the name of the Institutional Animal Care and Use Committee (IACUC) or other relevant ethics board that reviewed the study protocol, and indicate whether they approved this research or granted a formal waiver of ethical approval<br>• Include an approval number if one was obtained<br>• If the study involved *non-human primates*, add *additional details* about animal welfare and steps taken to ameliorate suffering<br>• If anesthesia, euthanasia, or any kind of animal sacrifice is part of the study, include briefly which substances and/or methods were applied<br><br>**Field Research**<br><br>Include the following details if this study involves the collection of plant, animal, or other materials from a natural setting:<br>• Field permit number<br>• Name of the institution or relevant body that granted permission	
**Data Availability**<br><br>Authors are required to make all data underlying the findings described fully available, without restriction, and from the time of publication. PLOS allows rare exceptions to address legal and ethical concerns. See the PLOS Data Policy and FAQ for detailed information.	Yes - all data are fully available without restriction

A Data Availability Statement describing where the data can be found is required at submission. Your answers to this question constitute the Data Availability Statement and **will be published in the article**, if accepted.<br><br>**Important:** Stating 'data available on request from the author' is not sufficient. If your data are only available upon request, select 'No' for the first question and explain your exceptional situation in the text box.<br><br>Do the authors confirm that all data underlying the findings described in their manuscript are fully available without restriction?	
**Describe where the data may be found in full sentences. If you are copying our sample text, replace any instances of XXX with the appropriate details.**<br><br>• If the data are **held or will be held in a public repository**, include URLs, accession numbers or DOIs. If this information will only be available after acceptance, indicate this by ticking the box below. For example: *All XXX files are available from the XXX database (accession number(s) XXX, XXX.).*<br>• If the data are all contained **within the manuscript and/or Supporting Information files**, enter the following: *All relevant data are within the manuscript and its Supporting Information files.*<br>• If neither of these applies but you are able to provide **details of access elsewhere**, with or without limitations, please do so. For example:<br><br>*Data cannot be shared publicly because of [XXX]. Data are available from the XXX Institutional Data Access / Ethics Committee (contact via XXX) for researchers who meet the criteria for access to confidential data.*<br><br>*The data underlying the results presented in the study are available from (include the name of the third party*	The data underlying the results presented in the study are available from Nagoya Institute of Clinical Pharmacology, http://www.nicp.jp/company/).

and contact information or URL).<br>• This text is appropriate if the data are owned by a third party and authors do not have permission to share the data.<br><br>* typeset	
Additional data availability information:	

Manuscript Click here to access/download;Manuscript;Manuscript.docx

Targeted vibratory therapy as a treatment for proprioceptive dysfunction: clinical trial in older patients with chronic low back pain

Yoshihito Sakai [1], Yoshifumi Morita [2], Keitaro Kawai [2], Jo Fukuhara [2], Tadashi Ito [3], Kazunori Yamazaki [4], Tsuyoshi Watanabe [1], Norimitsu Wakao [1], Hiroki Matsui [1]

[1] Department of Orthopaedic Surgery, National Center for Geriatrics and Gerontology, 7-430, Morioka-cho, Obu, Aichi Prefecture, Japan

[2] Department of Electrical and Mechanical Engineering, Graduate School of Engineering Nagoya Institute of Technology, Gokiso-cho, Showa-ku, Nagoya, Aichi Prefecture, Japan

[3] Three- Dimensional Motion Analysis Room, Aichi Prefectural Mikawa Aoitori Medical and Rehabilitation Center for Developmental Disabilities, 9-3 Koyaba, Kouryuji-cho, Okazaki, Aichi Prefecture, Japan

[4] Institutional Research Center, Aichi Mizuho College, 2-13 Shunko-cho, Mizuho-ku, Nagoya, Aichi Prefecture, Japan

Number of text pages of the entire manuscript: 28
Number of figures and tables: 1 figure, 4 tables

Correspondence author
Yoshihito Sakai
Email: jsakai@ncgg.go.jp

Short title: Targeted vibratory therapy for proprioceptive dysfunction

Abstract

Background

Proprioceptive function declines with age, leading to falls, pain, and difficulties engaging in activities of daily living among older adults. Although individuals with low back pain (LBP) have decreased lumbosacral proprioception in various postures, the mechanism by which reduced proprioceptive function causes LBP remains uncertain. Vibratory stimulation may enhance proprioceptive function; however, its efficacy as a treatment for LBP has not been investigated. This study investigated the feasibility of improving proprioceptive function and its effect on alleviating chronic LBP in older patients through targeted vibratory therapy (TVT) administration.

Methods

Older patients aged >65 years with nonspecific chronic LBP were recruited. TVT was performed by applying vibratory stimulation corresponding to the frequency of dysfunctional receptors for 1 min for 14 days to activate proprioceptors, with patients performing TVT three times daily at home. In cases with reduced proprioceptive function at multiple sites, the targeted treatment was aimed at the lowest frequency band value. Biological responses to vibratory stimulation targeting reduced proprioception in the trunk or lower legs were measured at sites with impaired function.

Findings

Overall, 56 patients with chronic LBP were enrolled, of whom 32 were recruited for treatment based on a proprioceptive dysfunction diagnosis. No adverse events were observed in any patient. After 2 weeks of TVT, numerical pain rating scale score improved to <3 in 78.1% of patients, 73.1% achieved a score ≤ 3, proprioceptive function improved in 81.3% in cases, and engagement in activities of daily living improved significantly.

Conclusion

TVT, involving vibratory stimulation at a receptor-specific frequency, demonstrated efficacy in

improving proprioception and alleviating LBP in older patients with impaired proprioceptive function without affecting non-targeted proprioceptors.

Introduction

The prevalence of chronic musculoskeletal pain is reported as 15·4% in the Japanese population, with low back pain (LBP) being the most common in men and women aged 75–84 years [1]. One cause of the increasing prevalence of LBP in older people is sarcopenia, an age-related condition that results in skeletal muscle mass reduction [2]. There is no consensus on the causal relationship between trunk muscle atrophy and LBP [3]; however, the fact that LBP is the most common chronic pain occurring in older adults presents a logical discrepancy. Regarding the relationship between skeletal muscles and LBP, the mechanism of segmental stability performed by local muscles, mainly the lumbar multifidus muscle, has long been considered important [4]. The significance of lumbar stability in regulating bipedal walking without LBP explains the rationale for investigating relationship among muscle spindle function, proprioception, and LBP [5]. This perspective highlights the significance of standing stability in the context of postural control and its relevance to LBP. Proprioception, a deep sensory function, detects the position of body parts, the state of muscle movement and contraction, resistance, and weight applied to the body. Proprioceptive feedback influences body movement and positional accuracy even without a visual sense, resulting in associative movements as somatosensory functions in human postural control. Proprioception encompasses signals from mechanoreceptors found in muscles, tendons, joints, and skin. In addition to muscle spindles in skeletal muscles, Golgi tendon organs in tendons, Meissner's corpuscles in the skin that sense touch, and Vater-Pacini corpuscles in

subcutaneous and periosteal tissue respond to vibration [6]. Vibratory stimulation of tissues elicits a proprioceptor response, leading to the perception of illusory movement [7]. Muscle-tendon vibration activates afferent nerve fibers, transmitted from the dorsal root of the spinal cord to the cerebrum, generating proprioception with different vibration frequencies for postural control [8].

Although numerous studies on proprioception and LBP have been published [2, 5], the mechanism by which reduced proprioceptive function causes LBP remains unclear. Systematic reviews with meta-analyses have yielded different conclusions regarding the causal relationship between LBP and proprioception: some indicate a relationship between proprioception and LBP [9, 10], some indicate insufficient evidence [11], others suggest a relationship with a small contribution [12], and some assert no relationship [13]. Therefore, we developed a diagnostic device to measure proprioceptive function in the trunk and lower limbs that corresponds to the vibration frequencies of all proprioceptors perceivable in humans [14], enabling accurate diagnosis of proprioceptive function. The vibration of skeletal muscles is known to stimulate proprioceptors and activate afferent nerve fibers. Furthermore, vibratory stimulation of the lumbar multifidus muscle in patients with LBP at a frequency corresponding to that of muscle spindles reportedly improves proprioceptive function [5]. Therefore, to enhance the existing literature on the connection between proprioception and LBP, it is useful to prospectively investigate potential changes in proprioceptive function. Consequently, this study investigated the feasibility of improving proprioceptive function and its effect on alleviating chronic

LBP in older patients through the administration of targeted vibratory therapy (TVT).

Methods

Study design and participants

Patients with non-specific chronic LBP were recruited from a single institute for the period April 2021 to March 2023. Eligible participants were older people aged over 65 years with LBP without sciatica for at least 6 months. We limited the study to patients aged over 65 years because postural responses to proprio-muscular inputs are affected by aging over 60 years [15]. Nonspecific LBP in this study was defined as follows: (1) LBP with numerical pain rating scale (NRS) score ≥ 3; (2) persistent pain localized below the costal margin and above the inferior gluteal folds; (3) absence of specific spinal pathologies such as infection, tumors, and vertebral fractures on both plain radiographs and lumbar magnetic resonance imaging (MRI); (4) no dominant leg pain caused by radicular and cauda equina disorders; (5) no significant instability such as spondylolysis, isthmic spondylolisthesis, and degenerative spondylolisthesis more than grade II; and (6) no previous lumbar and thoracolumbar spine surgery. All patients underwent Fall Risk Index evaluation [16], 10m walking speed measurement with normal gait, grip strength measurement (left and right average), five times chair rising test, whole-spine standing radiography, lumbar spine MRI, and dual-energy X-ray absorptiometry (DXA). Spinal sagittal alignment was evaluated by measuring lumbar lordosis (LL) from L1 to S1, lumbar range of motion (L-ROM) from L1 to S1, sagittal vertical axis (SVA), thoracic

kyphosis angle (TK), pelvic tilt (PT), pelvic incidence (PI), and pelvic incidence minus lumbar lordosis (PI-LL) in lateral view. MRI T2-weighted axial images were obtained to measure the cross-sectional area of the back muscles (sum of the left and right lumbar multifidus and erector spinae muscles) at the L4/5-disc level. Bone mineral density (T-score) at the L2–4 level and skeletal muscle mass index (SMI) (kg/m^2), calculated by dividing the skeletal muscle mass of the extremities by the square of height, were obtained using DXA. All patients were enrolled after confirming that their persistent pain could not be relieved using nonsteroidal anti-inflammatory drugs (NSAIDs) and opioid therapy (tramadol) for > 1 month. We excluded patients who could not stand unsupported, scored more than 6 points on the Fall Risk Index [16], or could not be evaluated using NRS owing to dementia.

This study was conducted according to the Declaration of Helsinki guidelines, and ethical approval was obtained from the University of Fujita Health University Certified Review Board (CRB4180003). This study was registered with the jRCT (jRCTs042200058) before the participants were enrolled, and all participants provided written informed consent.

Procedure

Diagnosis of proprioception

Diagnostic equipment developed by our research team was employed to assess proprioceptive function. (Fig 1) This device administers continuous vibrations ranging from 27 to 272 Hz separately to the trunk and lower legs in a sweeping manner. It gauges the biological response to the vibratory stimuli as an electrical signal indicating the displacement of the center of pressure (CoP) using a stabilometer

(T.K.K.5810, TAKEI Scientific Instruments Co., Ltd., Niigata, Japan) while the patient stands with their eyes closed. Vibratory stimuli, consisting of four vibrators (NSW1-205-8A, 5 W, 8 Ω, Aurasound, Inc., Santa Fe Springs, CA, USA), were applied to the bilateral gastrocnemius-soleus (GS) and lumbar multifidus (LM). The vibration section was divided into three evaluation section intervals of 15 seconds each ($ES_{1,2,3}$) determined according to the frequency of the vibratory stimulation corresponding to the proprioceptors. The subscript numbers of $ES_{1,2.3}$ are indicated as follows: 1: muscle spindles (lower frequency), from 30 to 53 Hz; 2: muscle spindles (higher frequency), from 56 to 100 Hz; and 3: Vater-Pacini corpuscle, from 140 to 250 Hz [17]. The sites where vibratory stimulation was applied were the GS and LM muscles, with each measurement time of 75 s consisting of the first 15 s without vibration (pre-section) and 60 s with vibration (vib-section). CoP was measured by applying local vibratory stimulation to the GS or LM muscles, with the patients' eyes closed. The root mean square (RMS) amplitude during the vibration of the GS and LM muscles was used as an indicator to evaluate the magnitude of the CoP in the anterior-posterior direction [18]. In this study, the modified RMS was calculated by the difference in CoP data between pre- and vib- sections as follows.

$$\mathrm{RMS}_{*}^{1} = \frac{\sqrt{\frac{1}{\mathrm{N}}\sum_{\mathrm{n}=\mathrm{n}_1}^{\mathrm{n}_2}\left\{\mathrm{Y}_{\mathrm{Vib}(*)}(\mathrm{n}) - \overline{\mathrm{Y}}_{\mathrm{pre}(*)}\right\}^2}}{\mathrm{RMS}_{*}^{\mathrm{pre}}}$$

$$\mathrm{RMS}_{*}^{2}=\frac{\sqrt{\frac{1}{\mathrm{N}}\sum_{\mathrm{n}=\mathrm{n}_3}^{\mathrm{n}_4}\left\{\mathrm{Y}_{\mathrm{Vib}(*)}(\mathrm{n})-\overline{\mathrm{Y}}_{\mathrm{pre}(*)}\right\}^2}}{\mathrm{RMS}_{*}^{\mathrm{pre}}}$$

$$\mathrm{RMS}_{*}^{3}=\frac{\sqrt{\frac{1}{\mathrm{N}}\sum_{\mathrm{n}=\mathrm{n}_5}^{\mathrm{n}_6}\left\{\mathrm{Y}_{\mathrm{Vib}(*)}(\mathrm{n})-\overline{\mathrm{Y}}_{\mathrm{pre}(*)}\right\}^2}}{\mathrm{RMS}_{*}^{\mathrm{pre}}}$$

The number N was equal to 300 because of the 15 s ES intervals, and the sampling frequency was 20 Hz. The RMSs were evaluated as the biological response to vibratory stimulation, indicating a proprioceptive function relative to the RMS value obtained without vibration. The greater the sway in each ES in the vibration section, the greater the RMS. A better proprioceptive function corresponds to a greater transition in the anteroposterior direction during vibratory application. Therefore, a higher RMS value indicates a superior proprioceptive function.

Fig 1. Diagnosis of proprioceptive function in the trunk and lower extremity
Vibratory stimulation of a wide range of frequencies from 27 Hz to 272 Hz is responsible for almost all proprioceptors and is administered to diagnose proprioceptive functions in older adults. The center of pressure (COP) displacement was continuously analyzed as a waveform using a stabilometer. Vibrations were applied to the bilateral lumbar multifidus and gastrocnemius soleus muscles and mechanical vibrations from low to high frequencies were applied continuously. The displacement of the COP during vibration at the trunk and calf was automatically recorded using the stabilometer to evaluate proprioceptor function.

For diagnosis of reduced proprioceptive function, RMS data from 81 young adults were used to identify the site and proprioceptors. An RNS value of -1 standard deviation (SD) relative to young adults was defined as proprioceptive dysfunction [18]. The RMS cutoff values for each site and frequency band were as follows.

RMS^{1}_{GS} (30–53 Hz): 1.01

RMS^{2}_{GS} (56–100 Hz): 1.02

RMS^{3}_{GS} (140–250 Hz): 0.69

RMS^{1}_{LM} (30–53 Hz): 0.85

RMS^{2}_{LM} (56–100 Hz): 0.84

RMS^{3}_{LM} (140–250 Hz): 0.65

TVT for proprioceptive dysfunction

TVT was performed on the portion of reduced function where the RMS was lower than the cutoff value. This was achieved by applying vibratory stimulation corresponding to the frequency of dysfunctional receptors for 1 min to activate the proprioceptors. The frequencies for treatment output were calculated based on a previous study [14]. Therapeutic vibratory stimulation lasted 60 s in each frequency band; the frequency rising and descending patterns were continuous for 30 s in the frequency band. The equipment was loaned to patients with proprioceptive dysfunction for use in either the trunk or lower extremities, covering both frequency bands, it was used for 14 days, with patients performing TVT three times daily at home. In cases with reduced proprioceptive function at multiple sites, the targeted treatment was aimed at the frequency band with the lowest RMS value. Patients were required to maintain a daily treatment diary, and any missing treatments of 10 % or more were excluded from the analysis. Based on a previous study that reported saturation of proprioceptive

function following vibratory stimulation was within 1 min [19], we set a treatment time of 1 min per TVT. Regarding treatment periods, a previous study administering opioids for chronic LBP demonstrated significant pain improvement after 2 weeks of medication [20]. Since we anticipated a treatment effect equivalent to pharmacotherapy for chronic LBP, we set the treatment period at 2 weeks. The clinical evaluation periods were pre-intervention, 2 weeks post-intervention, and 2 weeks following the conclusion of the intervention.

Outcome assessment

Changes in the intensity of LBP, disability caused by LBP, quality of life, mental quality, and central sensitization were assessed before the intervention and at the 2- and 4-week follow-ups. Pain intensity was measured on a NRS and scored from 0 (no pain) to 10 (worst possible pain). Disability resulting from LBP was evaluated using the Roland-Morris Disability Questionnaire (RDQ), which scored from 0 (no disability) to 24 (highest disability) [21]. Quality of life was assessed using the EuroQol-5D (EQ-5D) and EuroQol-visual analog scale, with a utility score anchored at 0 for death and 1 for perfect health, and 0 for the worst, and 100 for the best health, respectively [22]. The Geriatric Depression Scale-Short Form was used to assess depression among older adults. It is a 15-item yes-no self-report measure with a maximum score of 15, where higher scores represent more severe symptoms [23]. Central sensitization, an abnormal heightened sensitivity arising when the functionality of nociceptive pathways in the central nervous system is enhanced, was assessed using Central Sensitization

Inventory (CSI) with central sensitization-related items with total scores ranging from 0 to 100 [24].

Data management and statistical analysis

The planned sample size was calculated to provide 80% power for detecting a clinical difference in NRS scores of 1·7 points. This calculation assumed an SD of 3.1 aiming to achieve the same effects as 2-week opioid therapy for older patients with chronic LBP [20]. Twenty-nine patients were estimated to be required to give the trial 81.3% power to detect the difference in NRS using a paired test for the comparison of mean values at the 0.05 significance level.

We compared the baseline characteristics of patients with and without proprioceptive dysfunction. Improvements in LBP, ADL, and proprioceptive function were assessed by measuring changes before and after treatment. The statistical significance of the differences in pain intensity, ADL, and proprioceptive function was evaluated using the Wilcoxon signed-rank test.

All statistical analyses were performed using the EZR software (Saitama Medical Center, Jichi Medical University, Saitama, Japan). A p-value of less than 0.01 was considered to indicate a significant difference.

Results

Ultimately, 56 patients with chronic LBP (mean age 76.38±5.30 years; 28 males and 28 females) were enrolled, and all patients underwent proprioceptive function diagnostic evaluation. Among them, 32 patients were diagnosed with proprioceptive dysfunction in either the trunk or lower extremities, with

RMS values below the cutoff value, which is the standard for young, healthy participants. All 32 patients with chronic LBP were treated with TVT for 2 weeks to reduce proprioceptive dysfunction. The proportions and frequency bands of proprioceptive dysfunction were as follows: lumbar spine, 17 cases; lower limbs, 15 cases; 30-53 Hz, 10 cases; 56-100 Hz, 19 cases; and 140-250 Hz, 3 cases. Six patients had decreased proprioceptive function at multiple sites and frequency bands. No adverse events were observed in any patient. All patients completed 2 weeks of intervention and were followed-up and evaluated for 2 weeks after treatment completion.

A comparison between the 32 patients with impaired proprioceptive function and 24 patients with normal function is presented in Table 1. No significant differences were observed between the two groups in factors related to sarcopenia, such as grip strength, gait speed, and SMI. Furthermore, no differences were observed in clinical characteristics, including body composition, center-of-gravity test results without vibratory stimulation, and radiographic findings.

Table 1. Clinical characteristics in older patients with chronic low back pain with or without proprioceptive dysfunction

	Normal function N=24	Proprioceptive dysfunction N=32	p value
**RMS**	1.28 (0.34)	0.77 (0.15)	<0.0001
**Age (year)**	76.68 (5.80)	76.16 (5.00)	0.7251
**Sex (male : female)**	13:11	15:17	0.7875
**BMI (kg/m^2)**	23.73 (4.20)	24.57 (3.60)	0.4323
**Affected period of LBP (months)**	91.71 (48.76)	100.00 (59.64)	0.5804
**NRS**	6.48 (1.33)	6.31 (1.28)	0.6238
**RDQ**	9.94 (3.16)	8.79 (3.90)	0.2301
**EQ-5D**	0.66 (0.18)	0.62 (0.23)	0.4267
**EQ-VAS**	64.25 (15.60)	62.50 (14.74)	0.6723
**GDS**	2.88 (3.22)	3.88 (3.01)	0.2424
**CSI**	15.34 (9.71)	17.50 (10.10)	0.4224
**Fall risk index**	1.17 (1.55)	1.50 (1.69)	0.4519
**10 m walk speed (m/sec)**	1.10 (0.18)	1.09 (0.18)	0.8608
**Grip strength (kg)**	25.33 (7.47)	25.80 (7.69)	0.8204
**Chair rising test (sec)**	10.75 (4.93)	10.05 (2.90)	0.5102
**Total locus length (eye open) (mm)**	415.46 (125.28)	362.68 (104.70)	0.0919
**Total locus length (eye closed) (mm)**	603.01 (231.51)	544.39 (188.60)	0.3012
**Outer peripheral area (eye open) (mm^2)**	412.45 (224.91)	321.41 (166.56)	0.0873
**Outer peripheral area (eye closed) (mm^2)**	962.70 (774.97)	700.69 (482.10)	0.1257
**Romberg ratio (length)**	1.45 (0.37)	1.49 (0.28)	0.5692
**Romberg ratio (area)**	2.57 (1.90)	2.18 (1.11)	0.3447
**Antero-posterior RMS (eye open)**	5.13 (1.80)	4.46 (1.43)	0.1249
**Antero-posterior RMS (eye closed)**	6.93 (2.67)	6.50 (3.11)	0.5857
**Bone mineral density (L2-4 T-score)**	0.21 (2.22)	0.50 (2.53)	0.6594
**SMI (kg/m^2)**	6.61 (0.99)	6.67 (1.03)	0.8201
**Body fat ratio (%)**	31.91 (6.62)	33.07 (8.0)	0.5732
**Trunk muscle CSA**	1790.40 (581.08)	1554.39 (473.22)	0.0998
**LL (degree)**	29.92 (11.17)	25.00 (13.56)	0.1541
**L-ROM (degree)**	28.87 (13.47)	26.31 (11.78)	0.4578
**SVA (mm)**	69.96 (42.01)	72.07 (38.29)	0.8480
**TK (degree)**	38.12 (10.09)	37.00 (11.61)	0.7061
**PT (degree)**	26.92 (10.21)	26.47 (8.79)	0.8609
**PI-LL (degree)**	20.75 (12.63)	19.91 (13.90)	0.8164

Data are mean (SD).

RMS: root mean square, BMI: body mass index, LBP: low back pain, NRS: numerical pain rating

scale, RDQ: Roland-Morris Disability Questionnaire, EQ-5D: EuroQOL five dimensions questionnaire, EQ-VAS: EuroQOL visual analogue scale, GDS: Geriatric Depression Scale-Short Form, CSI: Central Sensitization Inventor, Total locus length: the sum of the path length of center of pressure, Outer peripheral area: the area that encloses the circumference of the stabilogram, SMI: skeletal muscle index, CSA: cross sectional area, LL: lumbar lordosis, L-ROM: lumbar ROM, SVA: sagittal vertical axis, TK: thoracic kyphosis, PT: pelvic tilt, PI-LL: pelvic incidence minus lumbar lordosis

During the 2-week TVT and 2-week post-intervention periods, 25 of 32 (78.1%) patients experienced NRS score improvement to less than 3 with intervention, and 11 (45.8%) of these patients experienced a worsened NRS score of 3 or greater 2 weeks after treatment completion. The mean NRS score after the 2-week TVT demonstrated significant improvement than before the intervention (p<0.0001). Relative to at the end of the intervention, the mean NRS score 2 weeks after the end of treatment increased from 2.6 to 3.6; however, this difference was not statistically significant (p=0.0162). Significant improvements in the RDQ, EQ-5D, and CSI assessments were observed after the 2-week TVT. No significant differences in ADL parameters were found between the intervention’s conclusion and 2 weeks after its completion (Table 2). The results of the pre- and post-intervention center-of-gravity tests and RMS values are presented in Tables 3 and 4, respectively. The results of the ordinary center-of-gravity test revealed no significant changes after the intervention or at the end of treatment. However, the mean RMS value of the TVT site, where the RMS value in each frequency band was below the cutoff value, demonstrated a significant improvement, increasing from a mean of 0.774 before treatment to 1.709 after 2 weeks of TVT (p<0.0001). Subsequently, the RMS value declined to

1.567 at 2 weeks after the end of treatment, with no significant difference from the value after 2 weeks of intervention (p=0.901). Among the 32 patients, 26 (81.3%) demonstrated improved RMS values above the cutoff values after 2 weeks of TVT and 19 (73.1%) patients achieved an NRS score of 3 or less.

Table 2. Results of targeted vibratory therapy for proprioceptive dysfunction

	Before treatment	2 weeks (treatment+)	4 weeks (treatment -)	p value [1] Mean difference 99% CI	p value [2] Mean difference 99% CI	p value [3] Mean difference 99% CI
**NRS**	6.31 (1.28)	2.61 (1.47)	3.61 (2.20)	<0.0001<br>-3.69<br>-4.49 to -2.90	0.0162<br>1.00<br>0.11 to 1.88	<0.0001<br>-2.70<br>-3.65 to -1.74
**RDQ**	9.94 (3.16)	4.50 (4.21)	4.22 (4.07)	<0.0001<br>-5.44<br>-6.69 to -4.19	0.5755<br>-0.28<br>-1.33 to 0.77	<0.0001<br>-5.72<br>-7.23 to -4.20
**EQ-5D**	0.66 (0.18)	0.83 (0.15)	0.85 (0.17)	<0.0001<br>0.17<br>0.09 to 0.25	0.4990<br>0.02<br>-0.04 to 0.08	<0.0001<br>0.18<br>0.11 to 0.26
**EQ-VAS**	64.25 (15.60)	71.56 (15.70)	67.16 (16.33)	0.0057<br>7.31<br>0.69 to 13.93	0.0201<br>-4.41<br>-9.14 to 0.33	0.3004<br>2.91<br>-4.79 to 10.60
**GDS**	2.88 (3.22)	2.13 (3.20)	1.69 (3.02)	0.0172<br>-0.75<br>-1.59 to 0.09	0.0910<br>-0.44<br>-1.10 to 0.23	0.0048<br>-1.19<br>-2.34 to -0.04
**CSI**	15.34 (9.71)	10.38 (6.75)	8.53 (6.99)	<0.0001<br>-4.97<br>-7.85 to -2.09	0.0495<br>-1.84<br>-4.20 to 0.51	0.0002<br>-6.81<br>-11.11 to -2.52

Data are mean (SD).
p value [1]: 2 weeks after treatment vs before treatment, p value [2]: 4 weeks vs 2 weeks after treatment, p value [3]: 4 weeks vs before treatment according to a Wilcoxon signed-rank test.
NRS: numerical pain rating scale for low back pain, RDQ: Roland-Morris Disability Questionnaire, EQ-5D: EuroQOL five dimensions questionnaire, EQ-VAS: EuroQOL visual analogue scale, GDS: Geriatric Depression Scale-Short Form, CSI: Central Sensitization Inventory

Table 3. Targeted vibratory therapy-related changes in swaying center of gravity without

vibration

	Before treatment	2 weeks (treatment+)	4 weeks (treatment -)	p value [1]<br>Mean difference<br>99% CI	p value [2]<br>Mean difference<br>99% CI	p value [3]<br>Mean difference<br>99% CI
**Total locus length (eye open) (mm)**	362.68<br>(104.70)	366.21<br>(121.62)	361.54<br>(120.66)	0.9553<br>3.53<br>-63.67 to 70.73	0.3694<br>-4.66<br>-35.67 to 26.35	0.9553<br>-1.13<br>-69.13 to 66.87
**Total locus length (eye closed) (mm)**	544.39<br>(188.60)	519.12<br>(188.16)	495.18<br>(190.97)	0.3498<br>-25.27<br>-139.75 to 89.22	0.1299<br>-23.95<br>-66.11 to 18.22	0.1347<br>-49.21<br>-160.49 to 62.07
**Outer peripheral area (eye open) (mm^2)**	321.41<br>(166.56)	352.44<br>(245.94)	376.02<br>(296.45)	0.8664<br>31.02<br>-98.86 to 160.91	0.8225<br>23.58<br>-71.88 to 119.04	0.5748<br>54.61<br>-108.70 to 217.91
**Outer peripheral area (eye closed) (mm^2)**	700.69<br>(482.10)	681.30<br>(418.30)	626.49<br>(473.08)	0.3595<br>-19.39<br>-295.67 to 256.89	0.1843<br>-54.81<br>-224.62 to 114.00	0.3595<br>-74.20<br>-348.70 to 200.29
**Romberg ratio (length)**	1.49<br>(0.28)	1.43<br>(0.30)	1.37<br>(0.29)	0.0979<br>-0.07<br>-0.28 to 0.14	0.3844<br>-0.05<br>-0.20 to 0.09	0.1098<br>-0.12<br>-0.32 to 0.08
**Romberg ratio (area)**	2.18<br>(1.11)	2.09<br>(0.86)	1.82<br>(0.99)	0.9297<br>-0.09<br>-0.84 to 0.65	0.2137<br>-0.27<br>-0.87 to 0.33	0.3694<br>-0.36<br>-1.09 to 0.37
**Antero-posterior RMS (eye open)**	4.46<br>(1.43)	4.96<br>(1.84)	5.00<br>(2.02)	0.1553<br>0.50<br>-0.40 to 1.40	0.8517<br>0.04<br>-0.72 to 0.81	0.2993<br>0.55<br>-0.67 to 1.76
**Antero-posterior RMS (eye closed)**	6.50<br>(3.11)	6.31<br>(2.24)	6.03<br>(2.36)	0.7577<br>-0.20<br>-2.00 to 1.61	0.5433<br>-0.28<br>-1.27 to 0.71	0.4716<br>-0.48<br>-1.96 to 1.00

Data are mean (SD).

The analysis of COP was performed using the following parameters, AP and ML range, sway velocity, length (cm), rectangle-area (cm^2), Romberg's quotient of length (%), and Romberg's quotient of rectangle-area (%).

p value [1]: 2 weeks after treatment vs before treatment, p value [2]: 4 weeks vs 2 weeks after treatment, p value [3]: 4 weeks vs before treatment according to a Wilcoxon signed-rank test.

COP: center of pressure, AP: antero-posterior, ML: medio-lateral

Table 4. Targeted vibratory therapy-related changes in proprioceptive function (RMS)

	Before treatment	2 weeks (treatment+)	4 weeks (treatment -)	p value [1]<br>Mean difference<br>99% CI	p value [2]<br>Mean difference<br>99% CI	p value [3]<br>Mean difference<br>99% CI
**Lower limb 30-53Hz**	1.500<br>(0.731)	1.735<br>(0.879)	1.557<br>(0.591)	0.2579<br>0.24<br>-0.29 to 0.76	0.5495<br>-0.17<br>-0.65 to 0.30	0.5372<br>0.06<br>-0.29 to 0.40
**Lower limb 56-100Hz**	1.449<br>(0.792)	1.851<br>(1.046)	1.699<br>(1.034)	0.1347<br>0.40<br>-0.20 to 1.01	0.3355<br>-0.15<br>-0.75 to 0.45	0.1937<br>0.25<br>-0.31 to 0.81
**Lower limb 150-250Hz**	1.350<br>(0.689)	1.769<br>(0.830)	1.657<br>(0.686)	0.0099<br>0.42<br>0.01 to 0.82	0.9702<br>-0.11<br>-0.50 to 0.28	0.0485<br>0.31<br>-0.09 to 0.70
**Lumbar spine 30-53Hz**	1.422<br>(0.710)	1.502<br>(0.680)	1.713<br>(1.156)	0.5434<br>0.08<br>-0.41 to 0.57	0.6006<br>0.21<br>-0.43 to 0.85	0.2699<br>0.29<br>-0.25 to 0.84
**Lumbar spine 56-100Hz**	1.388<br>(0.709)	1.745<br>(0.789)	1.797<br>(1.106)	0.0415<br>0.36<br>-0.09 to 0.80	0.4001<br>0.05<br>-0.62 to 0.72	0.0820<br>0.40<br>-0.21 to 1.02
**Lumbar spine 150-250Hz**	1.412<br>(0.754)	1.608<br>(0.830)	1.549<br>(0.778)	0.1184<br>0.20<br>-0.17 to 0.57	0.4377<br>-0.06<br>-0.53 to 0.42	0.7294<br>0.14<br>-0.31 to 0.58
**Most affected RMS**	0.774	1.709	1.567	<0.0001	0.4214	<0.0001

Data are mean (SD).
p value [1]: 2 weeks after treatment vs before treatment, p value [2]: 4 weeks vs 2 weeks after treatment, p value [3]: 4 weeks vs before treatment according to a Wilcoxon signed-rank test.
RMS: root mean square

Discussion

This is the first prospective study to evaluate the efficacy of TVT as a treatment for proprioceptive function, and it represents the first clinical study to establish a medical opinion on the relationship between proprioceptive function and LBP derived from an intervention study in patients with nonspecific chronic LBP. A unique aspect of this study is the evaluation of LBP improvement in older patients with impaired proprioceptive function, using an objective diagnosis of proprioceptive decline to target the impaired proprioceptors. Methods for activating proprioceptors through vibratory

stimulation to achieve proprioceptive improvement have been reported previously [5, 19]; however, this is the first attempt to use a therapeutic method for identifying receptors and sites with impaired function. The therapeutic vibratory approach, which targets specific proprioceptors with impaired function, effectively improves proprioception and prevents proprioceptive deterioration caused by harmful vibratory stimulation in healthy proprioceptors [25]. Therefore, it is essential to identify the specific proprioceptors and sites of impaired function before intervention.

Among older patients with chronic LBP, 57.1% exhibited reduced proprioceptive function, with dysfunction sites almost equally distributed between the trunk and lower extremities. This finding supports the research conclusions that chronic stress loading, arising from the overactivity of trunk proprioceptive function, can induce pain [26]. This occurs when the proprioceptive function of the lower limbs is reduced in addition to the trunk in older patients with chronic LBP [25]. Notably, patients diagnosed with proprioceptive dysfunction in chronic LBP displayed no distinctive clinical findings compared to those with normal proprioceptive function. This includes factors such as body composition or center-of-gravity sway, indicating that vibratory stimulation is essential for detecting proprioceptive pain.

All patients with chronic LBP and impaired proprioceptive function who underwent TVT experienced long-term pain and did not respond to 1 month of drug administration, with no anticipated carry-over effects or pain relief owing to natural history. The observation that a 2-week course of TVT resulted

in pain relief of 3.7 points (60%) on NRS, accompanied by notable enhancements in ADL in line with subjective pain assessment, is considered a favorable outcome associated with TVT. Among the 81.3% of older patients with chronic LBP whose proprioceptive function recovered to the level of younger healthy individuals, 73.1% had improved LBP with an NRS score of 3 or less. Overall, 59.4% (19 of 32) of patients demonstrated improvements both in proprioceptive function and LBP following to TVT. After the end of the treatment, the effect of TVT was maintained, both in pain and ADL. Proprioceptors, such as muscle spindles, exhibit specific responsive vibration frequency. Vibratory stimulation that matches the frequency of these proprioceptors activates afferent nerve fibers, thereby enhancing proprioceptive functions [7]. Given the reduced responsiveness to vibratory stimuli in impaired proprioceptors, the tendency of illusory movement is less likely to occur, resulting in impaired proprioception. A new concept in proprioceptive pain is thought to occur due to sensory-motor incongruity. The hypothesis that discordance between awareness of motor intention and muscle or joint proprioception results in pathologic chronic pain was proposed by Harris in 1999 [27]. Limited proprioceptive feedback leads to pathological pain in various parts of the body. Therapies that restore the integrity of cortical information processing for pain without underlying pathological causes have the potential to replace conventional analgesic treatments. The results of this study indicate an increase in the biological response to vibratory stimuli; specifically, proprioception is enhanced through the continuous application of the corresponding vibration specific to the impaired proprioceptors. The

significant improvements in proprioceptive function, pain intensity, and ADL associated with treatment, and their tendency to worsen after the end of the procedure, provided sufficient evidence that the effect of TVT on proprioception contributes to symptom improvement in older patients with chronic LBP.

The greatest concern regarding adverse reactions to TVT pertains to its effects on normal proprioceptors. Vibratory stimulation targeting dysfunctional receptors did not worsen the perturbation and effectively enhanced the responsiveness of the dysfunctional receptors. This indicates that the treatment affected the targeted proprioceptors without adversely affecting the overall center of gravity sway. Before this study, our research group evaluated the effects of therapeutic vibrations on proprioceptors. Vibratory stimulation with frequencies corresponding to the dysfunctional proprioceptors immediately improved their proprioceptive function. Simultaneously, the response of non-targeted receptors to vibratory stimulation was reduced, and proprioception worsened [28]. Lasting hyperactivation of proprioceptors continuously stimulates the reflex arc in the spinal cord, further inducing microglial activation, leading to the initiation and maintenance of pain [29]. Given that vibratory stimulation can cause harm to humans and induce LBP [28], we emphasize the importance of functional proprioception diagnosis in the development of vibration-based pain therapy. Vibratory therapy targeting dysfunctional proprioceptors is ideal for treating deteriorating proprioceptive function.

This study has some limitations. First, using a single arm without a control group obscured the interpretation of data integrity and validity regarding the treatment effects obtained. Establishing a placebo control group requires applying non-therapeutic vibratory stimulation in patients with chronic LBP and impaired proprioceptive function. However, as mentioned earlier, vibratory stimulation targeting proprioceptors other than dysfunctional ones should be avoided, as it could potentially exacerbate the deterioration in proprioceptive function. To address these limitations, we meticulously assessed the proprioceptive function of patients with chronic LBP and developed a treatment protocol that exclusively targeted the receptors with reduced function. This approach relies on assessments conducted at the site of proprioceptive dysfunction and the corresponding frequency band. Therefore, the effectiveness of TVT was examined by evaluating whether the improvement in proprioceptive function could be obtained consistently with beneficial changes in LBP. Second, there is controversy surrounding the appropriateness of the treatment duration setting. Although the patients demonstrated significant improvements in LBP and proprioceptive function after 2 weeks of treatment, a worsening trend was observed 2 weeks following the treatment completion. A comparison of different treatment periods and a study with a longer follow-up period are needed to determine the duration of treatment.

In conclusion, TVT, involving vibratory stimulation applied to impaired proprioceptors at a frequency corresponding to the receptor, improves proprioception and LBP in older patients with impaired proprioceptive function. Further sustained effects could be anticipated through modification of the

treatment period. This therapy presents a potential treatment modality for chronic pain.

Conclusion

TVT, involving vibratory stimulation applied to impaired proprioceptors at a frequency corresponding to the receptor, improves proprioception and LBP in older patients with impaired proprioceptive function. Further sustained effects could be anticipated through modification of the treatment period. This therapy presents a potential treatment modality for chronic pain.

Acknowledgments

We are grateful to all the patients who agreed to participate in the study, the staff who delivered the intervention, and the surgeons who helped with patient recruitment. We thank the Nagoya Institute of Clinical Pharmacology for providing an independent safety monitoring board and managing the data. We also appreciate the administrative assistance of Yayoi Sato, Junk Suzuki, and Miki Morita.

Financial and Material Support: This work was supported by National Center for Geriatrics and Gerontology (21-32). The funders were not involved in the design or conduct of the study, in the collection, management, analysis or interpretation of the data, or in the preparation, review or approval of the manuscript.

References

1. Nakamura M, Nishiwaki Y, Ushida T, Toyama Y. Prevalence and characteristics of chronic musculoskeletal pain in Japan. J Orthop Sci. 2011;16: 424-432. doi: 10.1007/s00776-011-0102-y

2. Sakai Y, Wakao N, Matsui H, Watanabe T, Iida H, Watanabe K. Clinical characteristics of geriatric patients with non-specific chronic low back pain. Sci Rep. 2022;12: 1286. doi: 10.1038/s41598-022-05352-2
3. Goubert D, Oosterwijck JV, Meeus M, Danneels L. Structural changes of lumbar muscles in non-specific low back pain: A systematic review. Pain Phys. 2016;19: E985-E1000.
4. Panjabi MM. The stabilizing system of the spine. part II. Neutral zone and instability hypothesis. J Spinal Disord. 1992;5: 390-396; discussion 397. doi: 10.1097/00002517-199212000-00002
5. Brumagne S, Cordo P, Lysens R, Verschueren S, Swinnen S. The role of paraspinal muscle spindles in lumbosacral position sense in individuals with and without low back pain. Spine. 2000;25:989-994. doi: 10.1097/00007632-200004150-00015
6. Riemann BL, Lephart SM. The sensorimotor system, part I: the physiologic basis of functional joint stability. J Athl Train. 2002;37: 71-79.
7. Roll JP, Vedel JP. Kinaesthetic role of muscle afferents in man, studied by tendon vibration and microneurography. *Exp Brain Res.* 1982;47: 177-190. doi: 10.1007/BF00239377
8. Polónyová A, Hlavacka F. Human postural responses to different frequency vibrations of lower leg muscles. Physiol Re.s 2001;50: 405-410.
9. Tong MH, Mousavi SJ, Kiers H, Ferreira P, Refshauge K, van Dieën J. Is there a relationship between lumbar proprioception and low back pain? A systematic review with meta-analysis. Arch

Phys Med Rehabil. 2017;98: 120-136 e2. doi: 10.1016/j.apmr.2016.05.016

10. Laird RA, Gilbert J, Kent P, Keating JL. Comparing lumbo-pelvic kinematics in people with and without back pain: A systematic review and meta-analysis. BMC Musculoskelet Disord. 2014;15: 229. doi: 10.1186/1471-2474-15-229

11. Rausch Osthoff AK, Ernst MJ, Rast FM, Mauz D, Graf ES, Kool J, Bauer CM. Measuring lumbar reposition accuracy in patients with unspecific low back pain. Systematic review and meta-analysis. Spine. 2015;40: E97-E111. doi: 10.1097/BRS.0000000000000677

12. Lin J, Halaki M, Rajan P, et al. Relationship between proprioception and pain and disability in people with non-specific low back pain: A systematic review with meta-analysis. Spine. 2019;44: E606-E617. doi: 10.1097/BRS.0000000000002917

13. Ghamkhar L, Kahlaee AH. Pain and pain-related disability associated with proprioceptive impairment in chronic low back pain patients: A systematic review. J Manipulative Physiol Ther. 2019;42: 210-217. doi: 10.1016/j.jmpt.2018.10.004

14. Nishio R, Ito Y, Morita Y, Ito T, Yamazaki K, Sakai Y. Investigation of the functional decline in proprioceptors for low back pain using the sweep frequency method. Appl Sci. 2019;9: 4988. doi: 10.3390/app9234988

15. Quoniam C, Hay L, Roll JP, Harlay F. Age effects on reflex and postural responses to propriomuscular inputs generated by tendon vibration. J Gerontol A Biol Sci Med Sci. 1995;50:

B155-B165. doi: 10.1093/gerona/50a.3.b155

16. Toba K, Okochi J, Takahashi T, Matsubayashi K, Nishinaga M, Yamada S et al. Development of a portable fall risk index for elderly people living in the community. Nihon Ronen Igakkai Zasshi. 2005;42: 346-352. doi: 10.3143/geriatrics.42.346
17. Ito Y, Kawai K, Morita Y, Ito T, Yamazaki K, Kato Y et al. Evaluation method of immediate effect of local vibratory stimulation on proprioceptive control strategy: A pilot study. Electronics. 2020;10: 341. doi: 10.3390/electronics10030341
18. Kawai K, Kato Y, Ito T, Yamazaki K, Fukuhara J, Sakai Y et al. Biological response and criterion value according to sweep frequencies of vibratory stimulation for the lower legs and trunk in healthy persons. Healthcare (Basel). 2023; 11: 2243. doi: 10.3390/healthcare11162243
19. Capicíková N, Rocchi L, Hlavacka F, Chiari L, Cappello A. Human postural response to lower leg muscle vibration of different duration. Physiol Res. 2006;55;Suppl 1: S129-S134. doi: 10.33549/physiolres.930000.55.S1.129
20. Sakai Y, Ito K, Hida T, Ito S, Harada A. Pharmacological management of chronic low back pain in older patients: A randomized controlled trial of the effect of pregabalin and opioid administration. Eur Spine J. 2015;24: 1309-1317. doi: 10.1007/s00586-015-3812-6
21. Roland M, Fairbank J. The Roland-Morris disability questionnaire and the Oswestry disability questionnaire. Spine. 2000;25: 3115-3124. doi: 10.1097/00007632-200012150-00006

22. Balestroni G, Bertolotti G. EuroQol-5D (EQ-5D): An instrument for measuring quality of life. Monaldi Arch Chest Dis. 2012;78: 155-159. doi: 10.4081/monaldi.2012.121

23. Yesavage JA, Brink TL, Rose TL, Lum O, Huang V, Adey M et al. Development and validation of a geriatric depression screening scale: A preliminary report. J Psychiatr Res. 1983;17: 37-49. doi: 10.1016/0022-3956(82)90033-4

24. Scerbo T, Colasurdo J, Dunn S, Unger J, Nijs J, Cook C. Measurement properties of the central sensitization inventory: a systematic review. Pain Pract. 2018;18: 544-554. doi: 10.1111/papr.12636

25. Boucher JA, Normand MC, Descarreaux M. Trunk isometric force production parameters during erector spinae muscle vibration at different frequencies. J Neuroeng Rehabil. 2013;10: 89. doi: 10.1186/1743-0003-10-89

26. Ito T, Sakai Y, Morita Y, Yamazaki K, Igarashi K, Nishio R et al. Proprioceptive weighting ratio for balance control in static standing is reduced in elderly patients with non-specific low back pain. Spine. 2018;43: 1704-1709. doi: 10.1097/BRS.0000000000002817

27. Harris AJ. Cortical origin of pathological pain. Lancet. 1999;354: 1464-1466. doi: 10.1016/S0140-6736(99)05003-5

28. Bovenzi M, Schust M, Mauro M. An overview of low back pain and occupational exposures to whole-body vibration and mechanical shocks. Med Lav. 2017;108: 419-433. doi:

10.23749/mdl.v108i6.6639

29. Yasui M, Menjyo Y, Tokizane K, Shinozawa A, Tsuda M, Inoue K, Kiyama H. Hyperactivation of proprioceptors induces microglia-mediated long-lasting pain in a rat model of chronic fatigue syndrome. J Neuroinflammation. 2019;16: 67. doi: 10.1186/s12974-019-1456-x

Figure
Click here to access/download;Figure;Fig 1.tif

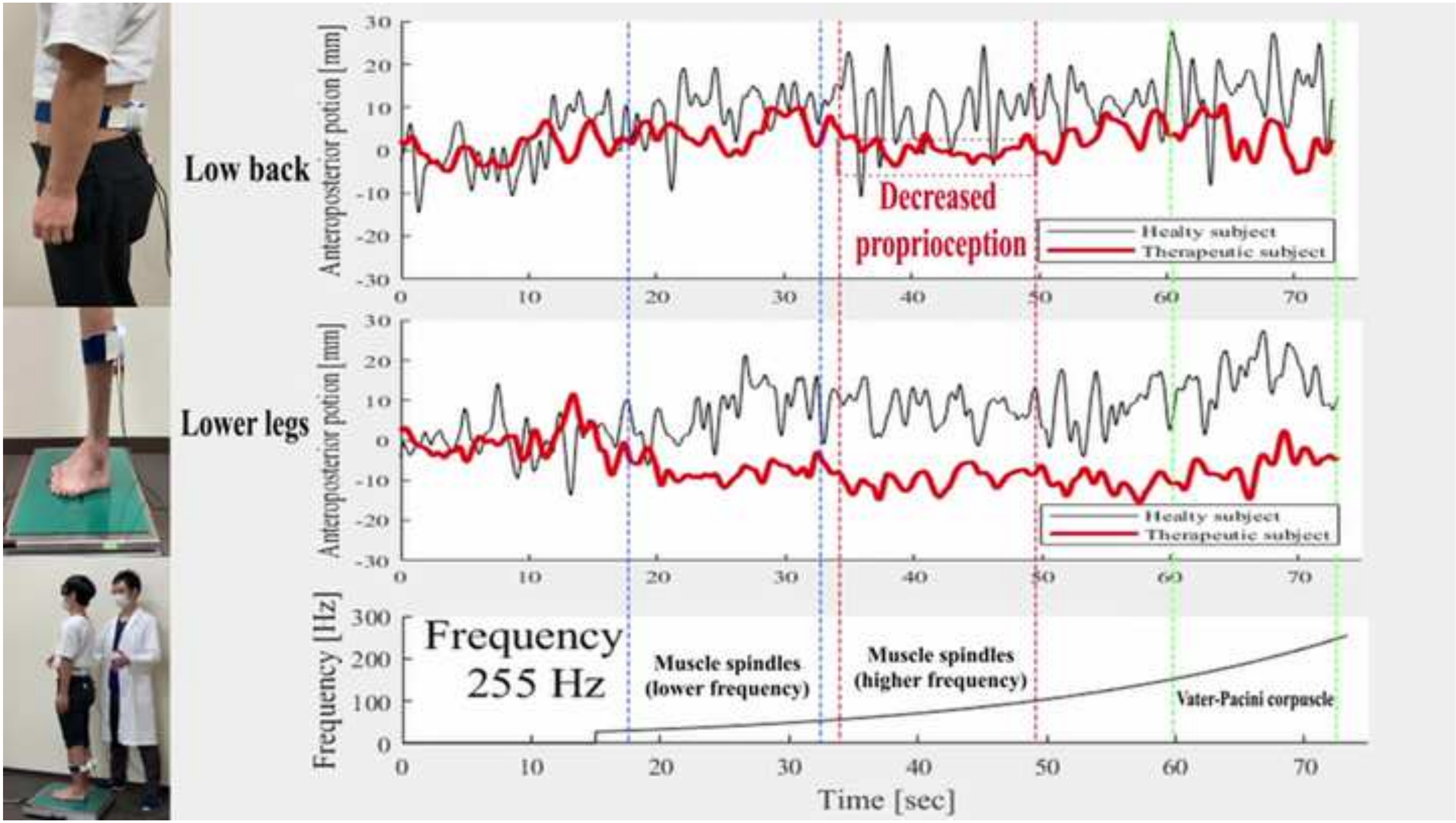

Click here to access/download
Supporting Information
Trial study protocol (Original).pdf

Click here to access/download
Supporting Information
Trial study protocol (English Translation) .pdf

Other

Click here to access/download
Supporting Information
trendstatement_TREND_Checklist.pdf

Click here to access/download
Other
Ethios approval (Original).pdf

Click here to access/download
Other
Ethics approval (English translation).pdf
