# Supplementary material for: Targeted vibratory therapy as a treatment for proprioceptive dysfunction: Clinical trial in older patients with chronic low back pain
Source: PLoS One. 2024 Jul 19;19(7):e0306898. doi: 10.1371/journal.pone.0306898 (PMC11259273; doi:10.1371/journal.pone.0306898)
Supplement: Attachment — Submitted filename: Response to Reviewers.docx [file pone.0306898.s005.docx]

**AUTHORS’ RESPONSES TO REVIEWERS’ COMMENTS**

We would like to thank the reviewers for their critique on our study, entitled “Targeted vibratory therapy as a treatment for proprioceptive dysfunction: clinical trial in older patients with chronic low back pain.” Their comments have helped us improve the quality of our work. The point-by-point responses to their comments are presented below. The revisions in our manuscript are highlighted in gray.

**Reviewer 1**

Abstract This does not really describe the study design and results in sufficient detail. Requires a major restructuring.

**Response:** We would like to thank the reviewer for evaluating our manuscript and for the insightful comments. In the Abstract, we have clarified the study design and added a comparison of proprioceptive dysfunction and normal function to clarify the two components of this study, as per the reviewer’s suggestion. The revised Abstract is as follows:

“Proprioceptive function declines with age, leading to falls, pain, and difficulties in performing activities of daily living among older adults. Although individuals with low back pain (LBP) exhibit decreased lumbosacral proprioception in various postures, the mechanism by which reduced proprioceptive function causes LBP remains uncertain. Vibratory stimulation may enhance proprioceptive function; however, its efficacy as a treatment for LBP has not been investigated. In this study, we investigated the feasibility of improving proprioceptive function and its effect on alleviating chronic LBP in older patients through targeted vibratory therapy (TVT) administration. Older patients aged >65 years with non-specific chronic LBP were recruited for a single-arm designed trial. TVT involved applying vibratory stimulation, matching the frequency of dysfunctional receptors, for 1 min daily over 14 days to activate proprioceptors, with patients performing TVT three times daily at home. In cases with reduced proprioceptive function at multiple sites, the targeted treatment was aimed at the lowest frequency band value. Biological responses to vibratory stimulation targeting reduced proprioception in the trunk or lower legs were measured at sites with impaired function. Overall, 56 patients with chronic LBP were enrolled, of whom 32 were recruited for treatment based on a proprioceptive dysfunction diagnosis, compared to 24 patients diagnosed as normal, with no significant differences observed in factors related to sarcopenia, and no clinical features in proprioception. No adverse events were observed in any patient. After 2 weeks of TVT, the numerical pain rating scale score improved to <3 points in 78.1% of patients, with 73.1% achieving a score ≤ 3 points. Proprioceptive function improved in 81.3% of cases, and engagement in activities of daily living improved significantly. TVT, involving vibratory stimulation at a receptor-specific frequency, demonstrated efficacy in improving proprioception and alleviating LBP in older patients with impaired proprioceptive function without affecting non-targeted proprioceptors.”

P213 line 213-217 This section needs rephrasing.

**Response:** We have made the following revisions in accordance with the reviewer’s suggestion.

“The aim was to demonstrate the same clinical difference in the NRS scores as those achieved following 2-week opioid therapy for older patients with chronic LBP [22]. This suggested a difference in the NRS of 1.7 (SD, 3.1) points. For a paired t-test with a two-sided significance level of 0.05 and 80% power, requires 29 patients needed to be recruited.” (Lines 222-225)

P213 line 219-220 This does not describe how the statistical comparisons of Tables 2, 3 & 4 were conducted. The sample size calculation depends on the use of the Student’s t-test so this needs to be mentioned here. Also, CIs are used in the tables but there is no mention here.

**Response:** In Table 2, the Mann–Whitney U test was used for the two-group comparison in the presence of proprioceptive dysfunction, and a description of CI was added. The following changes were made in the “Data Management and Statistical Analysis” subsection:

“The aim was to demonstrate the same clinical difference in the NRS scores as those achieved following 2-week opioid therapy for older patients with chronic LBP [22]. This suggested a difference in the NRS of 1.7 (SD, 3.1) points. For a paired t-test with a two-sided significance level of 0.05 and 80% power, requires 29 patients needed to be recruited.

We compared the baseline characteristics of patients with and without proprioceptive dysfunction using the Mann–Whitney U test. Improvements in LBP, activities of daily living (ADLs), and proprioceptive function were assessed by measuring changes before and after treatment. The statistical significance of the differences in pain intensity, ADLs, and proprioceptive function was compared between pre-treatment and 2 weeks after treatment, 2 weeks and 4 weeks after treatment, and pre-treatment and 4 weeks after treatment using the Wilcoxon signed-rank test. The results are presented as mean differences for continuous outcomes with 99% confidence intervals (CIs).” (Lines 222-232)

Page13 line 226 More useful to give the minimum, median and maximum ages concerned.

**Response:** Please note that the minimum, median, and maximum ages were added. The revised part is as follows:

“Ultimately, 56 patients with chronic LBP (mean age, 76.38±5.30 years; minimum, 67 years; median, 76 years; maximum, 87 years; sex, 28 male and 28 female individuals) were enrolled, and all patients underwent proprioceptive function diagnostic evaluation.” (Lines 236-238)

Page 15 Table 1 This table is far too detailed. It would help if the number of decimal places used was reduced. For example, for total focus length (eye open) replace ‘415.46 (125.28)’ by ‘416 (125)’.

**Response:** In accordance with the reviewer's suggestion, the number of digits in Table 1 was reduced to zero by rounding off the decimal point in values with more than three digits.

Not clear what is meant by ‘RMS’?

**Response:** A description of RMS can be found in line157 of the revised manuscript (Methods section). For clarity, we have also added the following sentence to table footnotes.

“The root mean square (RMS) was calculated by the difference in CoP data between the pre- and vib- sections. (See Diagnosis of proprioception).”

Table 1 & elsewhere Reduce p-values to 2 significant figures. For example,’ 0.7251 to 0.73’ and ‘0.0919 to 0.092’.

**Response:** We have made the appropriate revisions in Table 1-4.

Suggest the order within current headings of Columns 5, 6, 7 should be ‘difference, 99%CI, then p-values’ as the magnitude of the differences is the principal focus.

**Response:** We agree with this comment, and have corrected the order of Columns 5, 6, and 7 in all of Table 2, 3, and 4 from ↑ to difference, 99%CI, and p value.

Needs to indicate how many patients are concerned with each variable – is it always 32?

**Response:** The number of patients for each variable is always 32, as all 32 patients treated were evaluated. No patient dropped out during the study.

Not sure if Column 6 is worth reporting although the magnitude of that difference between weeks 2 and 4 weeks deserves comment. Do they indicate that (more-or-less) the value of the target therapy plateau’s after week 2? In any event, I suggest the statistical test results are omitted.

**Response:** We would like to thank the reviewer for the valuable suggestions. Following a 2-week treatment period, we have undertaken the assessment of treatment persistence by temporarily halting TVT and subsequently evaluating the patients’ condition after a further 2 weeks. Thus, we opted to describe the results of the study, as we believe that demonstrating that patients maintained improved condition post-treatment, despite experiencing slight deterioration due to the treatment interruption, could serve as justification for a reasonable TVT treatment period. The study protocol also calls for an evaluation at 2 weeks after the end of treatment, and we think that it is desirable to include this information without omitting it. We appreciate the reviewer’s comments, and we will use them as a reference for future protocol development.

**Reviewer 2**

Please, find my comments and suggestions in the attached file.

P6 line81 I suggest adding references here. Some suggestions are:

1: van Heuvelen MJG, Rittweger J, Judex S, Sañudo B, Seixas A, Fuermaier ABM, Tucha O, Nyakas C, Marín PJ, Taiar R, Stark C, Schoenau E, Sá-Caputo DC, Bernardo-Filho M, van der Zee EA. Reporting Guidelines for Whole-Body Vibration Studies in Humans, Animals and Cell Cultures: A Consensus Statement from an International Group of Experts. Biology (Basel). 2021, 27;10(10):965. doi: 10.3390/biology10100965.

2: Sá-Caputo D, Taiar R, Martins-Anjos E, Seixas A, Sartório A, Sanudo B, Sonza A, Amaral V, Lacerda A, Gomes-Neto M, Moura-Filho O, Oliveira L, Bachur J, Bernardo-Filho M. Does the mechano-biomodulation vibration lead to biological responses on human beings. Series on Biomechanics, 2023, 37 (2): 3-17. doi: 10.7546/SB.01.02.2023

I am suggesting an inclusion of a reference, that is the guidelines about the utilizations of mechanical vibrations in health sciences.

**Response:** We would like to thank the reviewer for evaluating our manuscript and for the insight comments. Although this study is not a report on WBV treatment, we believe that it should be conducted in accordance with these guidelines in terms of vibration to the human body. We have published the information on the vibration used in this study in thesubsection "TVT for proprioceptive dysfunction" in the Methods section, according to the suggested Reference #1. The added part is as follows:

“The vibratory intervention programs of this trial are structured according to previously published guidelines for whole-body vibration studies in information about the device and vibration [20]. The device used in this therapeutic trial is non-commercial (self-built), and uses the same vibrators as the diagnostic devise described above. Briefly, the device is 60 mm * 75 mm * 3 mm large, has a mass of 52.3 g, and can deliver sinusoidal (deviation <5%) vibration per vibrator. The device can produce vibrations with peak-to-peak displacement up to 1.6 mm within a frequency range of 30–53Hz, 56–00Hz, and 140–250Hz. The vibrator produced sinusoidal accelerations primarily in the vertical component, with minimal variation in acceleration direction throughout each sinusoidal cycle.” (Lines 183-191)

P7 line 102 Defne it.

**Response:** Please note that the name of the facility was added.

“a single institute (National Center for Geriatrics and Gerontology).”

P7 line 106 add reference

**Response:** Please note that we have added a relevant references.

Reference

Shafshak TS, Elnemr R. The visual analogue scale versus numerical rating scale in measuring pain severity and predicting disability in low back pain. J Clin Rheumatol. 2021;27: 282-285. doi: 10.1097/RHU.0000000000001320.

P19 line 223 Put all "p" related to statistical analysis in italic format, here abd throughout the manuscript.

**Response:** Please note that we have used italics when presenting the p-values.

P23 line 377-379 I suggest considering these sentences as the strengths of the study. Use a single paragraph with these statements.

**Response:** We would like to thank the reviewer for the suggestion. Please note that we have moved the sentence from Lines 377–380 before the limitation and used a single paragraph to describe the strengths of this study. The added part is as follows:

“The greatest strength of this study lies in its rigorous diagnosis of proprioceptive dysfunction and targeted identification of impaired proprioceptors, which allowed for the precise application of vibratory stimulation without adverse effects. This TVT effectively improved proprioception and alleviated chronic LBP in older patients with impaired proprioceptive function. With the potential for sustained effects through treatment period modifications, TVT represents a promising modality for chronic pain management”. (Lines 403-408)

**Reviewer 3**

The current study aimed to investigate the feasibility of improving proprioceptive function and its effect on alleviating chronic LBP in older patients through targeted vibratory therapy (TVT) administration. The approach is original. The manuscript reads smoothly and is easy to understand. The aims, scope, and results of the study are clearly stated. I have very much enjoyed reading this paper. I find it interesting and clearly written and satisfying also all the other publication criteria of the “PLOS ONE”. The study provides a very valuable addition to this line of research and adds relevantly to the subject with additional original findings. I thus find that this paper definitively delivers results that will surely be of interest to the readership of the “PLOS ONE”. I recommend the publication of this paper after revision. The authors must develop the limitations of the study. The authors must use REF from serious journals and indexed. I recommend the addition of the following references that will increase the methodology and discussion sections that appears still poor. They are the guideline of this area of this research and must be used like a reference of methodology.

• Wuestefeld A, FuermaierABM, Bernardo-Filho M, da Cunhade Sa ́-CaputoD, Rittweger J, Schoenau E, et al. (2020). Towards reporting guidelines of research using whole-body vibration as training or treatment regimen in human subjects—A Delphi consensus study. PLoSONE, 15(7):e0235905 <https://doi.org/10.1371/journal.pone.0235905>
• Acute Effects of Whole-Body Vibration on the Pain Level, Flexibility, and Cardiovascular Responses in Individuals With Metabolic Syndrome. Dose-Response: October-December 2018:1-9. <https://doi.org/10.1177/1559325818802139>.
• Relevance of Whole-Body Vibration Exercises on Muscle Strength/Power and Bone of Elderly Individuals. DOI <https://doi.org/10.1177/1559325818813066>
• Whole-body vibration improves the functional parameters of individuals with metabolic syndrome: An exploratory study. DOI <https://doi.org/10.1186/s12902-018-0329-0>
• Do whole body vibration exercises affect lower limbs neuromuscular activity in populations with a medical condition? A systematic review. DOI <https://doi.org/10.3233/RNN-170765>
• Whole-body vibration improves the functional parameters of individuals with metabolic syndrome: An exploratory study. <https://doi.org/10.1186/s12902-018-0329-0>
• Potential application of whole body vibration exercise for improving the clinical conditions of covid-19 infected individuals: A narrative review from the world association of vibration exercise experts (wavex) panel DOI 10.3390/ijerph17103650
• Whole-body vibration improves the functional parameters of individuals with metabolic syndrome: An exploratory study 10.1186/s12902-018-0329-0
• Attitudes to knee osteoarthritis and total knee replacement in Arab women: A qualitative study 10.1186/1756-0500-6-406
• Moreira-Marconi, E. et al. Evaluation of the temperature of posterior lower limbs skin during the whole body vibration measured by infrared thermography: Cross-sectional study analysis using linear mixed effect model (2019) PLoS ONE, 14 (3), art. no. e0212512

**Response:** We would like to thank the reviewer for evaluating our manuscript and for the insightful comments. Please note that we have added the following statement regarding vibration as a treatment in Methods section, as the WBV guidelines are needed to clarify the information on vibration. The following references were used for the guideline, as they are more recent.

van Heuvelen MJG, Rittweger J, Judex S, Sañudo B, Seixas A, Fuermaier ABM, Tucha O, Nyakas C, Marín PJ, Taiar R, Stark C, Schoenau E, Sá-Caputo DC, Bernardo-Filho M, van der Zee EA. Reporting Guidelines for Whole-Body Vibration Studies in Humans, Animals and Cell Cultures: A Consensus Statement from an International Group of Experts. Biology (Basel). 2021, 27;10(10):965. doi: 10.3390/biology10100965.

The added part is as follows:

“The vibratory intervention programs of this trial are structured according to previously published guidelines for whole-body vibration studies in information about the device and vibration [20]. The device used in this therapeutic trial is non-commercial (self-built), and uses the same vibrators as the diagnostic devise described above. Briefly, the device is 60 mm * 75 mm * 3 mm large, has a mass of 52.3 g, and can deliver sinusoidal (deviation <5%) vibration per vibrator. The device can produce vibrations with peak-to-peak displacement up to 1.6 mm within a frequency range of 30–53Hz, 56–00Hz, and 140–250Hz. The vibrator produced sinusoidal accelerations primarily in the vertical component, with minimal variation in acceleration direction throughout each sinusoidal cycle.” (Lines 183-191)

To enrich the Discussion section, we have used two suggested references and added a possible mechanism for improving pain by applying vibration to the muscles. The added part is as follows:

“There have been several reports of whole-body vibration (WBV) as a mechanism by which vibration stimulation of skeletal muscles contributes to pain reduction. Vibratory stimulation has been suggested to increase muscle flexibility by activating the Ia inhibitory interneurons of the antagonist muscle following vibration [32]. Additionally, it is proposed that vibration may induce changes in intramuscular coordination ,thereby reducing the braking force around the joints in the lower back [33]. One effect on proprioception is the activation of inhibitory circuits in the dorsal horn of the spinal cord, facilitated by the transmission from proprioceptive receptors through sensory fibers type Aβ. These circuits subsequently diminish the pain input to C fibers, resulting in pain reduction [34]. Moreover, increased secretion of myokine, an anti-inflammatory cytokine, has been reported as a response of skeletal muscles to WBV [35], and it may be expected that the contraction of skeletal muscles may induce analgesia through exercise. While the mechanism behind the pain-relieving effect of vibration stimulation on skeletal muscles remains inconclusive, it is important to note that the vibration stimulation used in this study was not WBV; however, the vibration device used in this study was operated in accordance with the guidelines on WBV [20]. The fact that TVT was effective in improving proprioceptive function and LBP at the same time in the present study supports the relationship between proprioception and LBP.” (Lines 387-402)
