# Supplementary material for: Targeted vibratory therapy as a treatment for proprioceptive dysfunction: Clinical trial in older patients with chronic low back pain
Source: PLoS One. 2024 Jul 19;19(7):e0306898. doi: 10.1371/journal.pone.0306898 (PMC11259273; doi:10.1371/journal.pone.0306898)
Supplement: Attachment — Submitted filename: Response to Reviewers.docx [file pone.0306898.s006.docx]

Response to Reviewers

We appreciate the reviewers for the critique on our study titled; “Targeted vibratory therapy as a treatment for proprioceptive dysfunction: clinical trial in older patients with chronic low back pain.” . We have revised the manuscript according to the reviewer’s comments. Revisions are highlighted in gray in the attached manuscript.

Reviewer #4: First of all, I want to congratulate you for the work you have done. I communicate a series of suggestions that could improve the quality of your work:

- Introduction: Although it is not necessary to have a structured abstract, we recommend rewriting it in order to highlight and make each of the sections clear. I also recommend reviewing the writing as it can be improved.

Answer: Thank you for the suggestion. Accordingly, the abstract has been structured to clearly demarcated each of the sections and the language of the abstract has been rechecked.

- Line 47-48: Explain this sentence, it’s not understood.

Answer: Apologies for the lack of clarity before. Lines 47-49 “Biological responses to vibratory stimulation targeting reduced proprioception in the trunk or lower legs were measured at sites with impaired function.” have now been revised as follows:

LBP and proprioceptive function were evaluated at 2 weeks after TVT and at 2 weeks after the end of TVT in patients with declined proprioception in the trunk or lower extremities.

- Line 63: Fixe double spacing.

Answer: Thank you for pointing this out. The manuscript has been double-spaced throughout.

- Line 100: Explain if participants were asked to sign the informed consent and if it was explained to them.

Answer: Thank you for this advice. The following text has been added.

Participants were briefed on the study and each of them provided informed consent.

- Line 289, 302 and 317: Please, explain under the table what ‘treatment+’ and ‘treatment-‘ mean.

Answer: Apologies for the lack of clarity before. The following explanatory text has been added.

treatment+ indicates 2 weeks after treatment and treatment- indicates 2 weeks after the end of treatment.
